# Dynamic shift in the dominant transmission route of clade Ib monkeypox virus across networks with sexual and nonsexual contacts

**DOI:** 10.1126/sciadv.aec1931

**Published:** 2026-04-01

**Authors:** Fuminari Miura, Ka Yin Leung, Maria Xiridou, Marten van Antwerpen, Nicola Low, Niel Hens, Emmanuel Hasivirwe Vakaniaki, Jacco Wallinga

**Affiliations:** ^1^ Centre for Infectious Disease Control, National Institute for Public Health and the Environment, Bilthoven, Netherlands.; ^2^ Center for Marine Environmental Studies, Ehime University, Matsuyama, Japan.; ^3^ Institute of Tropical Medicine, Nagasaki University, Nagasaki, Japan.; ^4^ Institute of Social and Preventive Medicine, University of Bern, Bern, Switzerland.; ^5^ Data Science Institute, I-BioStat, Hasselt University, Hasselt, Belgium.; ^6^ Centre for Health Economic Research and Modelling Infectious Diseases, Vaccine and Infectious Disease Institute, University of Antwerp, Antwerp, Belgium.; ^7^ Institut National de Recherche Biomédicale (INRB), Kinshasa, Democratic Republic of the Congo.; ^8^ Department of Clinical Sciences, Institute of Tropical Medicine, Antwerp, Belgium.; ^9^ Department of Microbiology, Immunology and Transplantation, KU Leuven, Leuven, Belgium.; ^10^ Department of Biomedical Data Sciences, Leiden University Medical Center, Leiden, Netherlands.

## Abstract

The intensifying outbreaks of the novel monkeypox virus clade Ib in the Democratic Republic of the Congo have raised global concern about the potential for wider epidemic spread. Some clade Ib mpox outbreaks have shown a distinct transmission pattern in which transmission associated with both sexual and nonsexual contacts coexist. Here, we characterize these outbreaks in a network epidemic model, which incorporates sexual and nonsexual contacts, and project age- and route-specific transmission potentials under a wide range of scenarios. Our analyses suggest that the dominant route of transmission may shift over time from sexual to nonsexual contacts, which leads to larger epidemics. The age groups contributing most to overall infections and mortality also change over time, suggesting that target groups for intervention should be adjusted accordingly. For countries at risk of travel-associated mpox outbreaks, these findings highlight the importance of monitoring evolving monkeypox virus transmission patterns and interacting transmission routes to support timely and effective control measures.

## Teaser

During an mpox outbreak, the dominant route of transmission and optimal target groups for intervention may shift over time.

## INTRODUCTION

The emergence of clade Ib monkeypox virus (MPXV) in the Democratic Republic of the Congo (DRC) in late 2023 has raised renewed global concern about the potential for wider epidemic spread (*1*). In August 2024, the World Health Organization declared a second Public Health Emergency of International Concern about mpox in response to escalating outbreaks in Central and East Africa, driven by this novel subclade (*2*). Initially identified in Kamituga health zone, South Kivu province, clade Ib has since spread to multiple neighboring countries and travel-associated cases have been reported in Europe, the Americas, and Asia (*3*). Although sustained transmission of clade Ib outside Africa has not been confirmed, as of June 2025, the combination of uncertain epidemiology, likely underdetection, and limited control capacity in affected regions makes further national and international spread possible (*4*). Risk assessments are underway in Europe and other regions to evaluate the potential impact of future introductions and to guide preparedness planning (*5*, *6*).

The transmission dynamics of clade I MPXV are changing. MPXV is historically a zoonotic pathogen, with clade Ia circulating through animal-to-human and subsequent limited human-to-human transmission (*7*). In contrast, the outbreaks of clade Ib have been characterized by sustained human-to-human transmission via close contact either within households and communities (“nonsexual” route) or in association with sexual encounters (“sexual” route). Studies based on data collected in South Kivu in 2024 to 2025 suggest that key epidemiological parameters for clade Ib MPXV vary by transmission route; for instance, incubation periods and serial intervals may be shorter for transmission via sexual contacts than via nonsexual contacts (*8*, *9*). However, secondary attack risks (SARs), reproduction numbers, and other key parameters remain poorly quantified, especially in different age groups. Case fatality ratios (CFRs) for clade I MPXV have historically ranged from 1 to 10% (*7*, *10*), with recent crude estimates for clade Ib ranging from 0.2 to 0.5% (*3*). These CFRs appear to be higher in young children and lower in adults, although differences across age and clade may reflect variation in surveillance capacity or access to clinical care.

Capturing the dynamics of mpox within and across networks of sexual and nonsexual contacts may help to design efficient control strategies. Clinical and epidemiological investigations suggest that the initial phase of the clade Ib outbreak in Kamituga was driven by transmission via sexual contacts between women and men (*1*, *11*), with subsequent transmission from person to person via close physical contacts in the household or community among children under 15 years old (*12*). Following the spread of clade Ib MPXV to Burundi, the age and sex distribution of mpox cases there also suggests spread through both sexual and nonsexual contacts (*13*). This pattern contrasts with the 2022 global outbreak of clade IIb mpox, where transmission via sexual contacts occurred mainly between men who have sex with men and transmission within the household to younger age groups was minimal (*14*, *15*). In Sierra Leone, however, where a large clade IIb MPXV outbreak started in 2025, the distribution of cases also suggests transmission via both sexual and nonsexual contacts (*16*).

Multiple transmission routes, including through sexual contact, have been described for emerging infectious diseases, such as Ebola (*17*) and Zika (*18*). Existing mathematical models describing transmission via both sexual and nonsexual contacts often simplify transmission via sexual contacts by using approximated network structure derived from the assumption that individuals only contact each other in one-off encounters, for Zika and Ebola (*19*–*21*) and mpox (*22*). Network models that explicitly capture sexual contact networks can represent more realistic characteristics (*23*–*25*). Earlier studies have analyzed multiroute transmission (*26*) and demonstrated that asymmetric sexual transmission can create a two-stage spread on sexual networks, starting within one sex group and later propagating to others (*27*, *28*). These approaches do not explicitly capture age structure or temporal shifts in dominant transmission routes. The use of these approaches is hindered by the complexity and the challenges in linking model structure to epidemiological data collected during outbreaks (*29*, *30*), particularly when disease outcomes and intervention priorities vary across age groups.

To address these gaps, we developed a network epidemic model tailored to clade Ib mpox, using the Kamituga outbreak as an example, where multiple, age-structured contact routes of human-to-human transmission have interacted and evolved over time. Building on prior frameworks (*23*, *24*, *31*), our model incorporates two types of contacts: sexual and nonsexual; nonsexual contacts are defined as age-specific contacts and are parameterized by a contact matrix (*32*). Sexual contacts are modeled to occur within pairs of sexually active individuals (partnerships), forming a static sexual network according to the distribution of the number of sexual partners.

The primary objective of this study is to determine how the dominant route of human-to-human transmission evolves over time, using epidemiological data that can be collected during outbreaks of clade Ib MPXV. We project age- and route-specific contributions to transmission at different epidemic stages as well as epidemic sizes by transmission route. We also show how the model can be used to identify which subgroups would be most effective to target with interventions, based on their projected contributions to onward transmission and mortality (*32*–*34*). Last, although motivated by ongoing clade Ib outbreaks, the proposed framework aims to be broadly applicable to infections transmitted through both sexual and nonsexual contacts. In particular, we explore a wide range of scenarios to reflect differential epidemiological and behavioral conditions across mpox outbreak settings, not necessarily limited to clade Ib. This makes our study generic and relevant for supporting risk assessment not only in currently affected countries such as the DRC but also in countries at risk of future mpox importation, including those outside the African region.

## RESULTS

### Model overview

We developed a network epidemic model with two routes of transmission via close contact—sexual and nonsexual—including age differences, while simplifying by ignoring sex differences. The sexual contacts were represented using a configuration network construction (*35*), while the nonsexual contacts were modeled through age-dependent random mixing using an age-structured contact matrix (*36*). Infection could be transmitted through either route at distinct transmission rates. The model is described by a set of ordinary differential equations (ODEs). We analyzed this ODE system to characterize the epidemic behavior. Full details are provided in Materials and Methods and the Supplementary Materials.

### Projected transmission potential of each transmission route

To project route- and age-specific transmission potentials, we computed a projection matrix that has the number of new infections produced by each age group and route of transmission as its elements [i.e., the next-generation matrix (NGM) (*32*, *37*)]. The NGM needs to be subdivided into four submatrices, which describe transmission from one route of an index case to another route of secondary cases (via either sexual or nonsexual contact, indexed as s and ns, respectively), rather than only the route of transmission of secondary cases (see the detailed derivation in the Supplementary Materials) (*23*, *24*, *26*, *31*). As the expected number of secondary cases through one route of transmission depends on the route of transmission of the index case, it is not possible to further reduce the NGM. Each submatrix can be summarized as a route-to-route specific reproduction number; $R_{\text{s,ns}}$ represents the expected number of new infections via sexual contacts transmitted by a case who acquired infection via nonsexual contact (“from-nonsexual-to-sexual” reproduction number) in the absence of other transmission routes. Similarly, $R_{s,s}$, $R_{\text{ns,s}}$, and $R_{\text{ns},\text{ns}}$ correspond to reproduction numbers for “from-sexual-to-sexual,” “from-sexual-to-nonsexual,” and “from-nonsexual-to-nonsexual,” respectively.

**Fig. 1.**
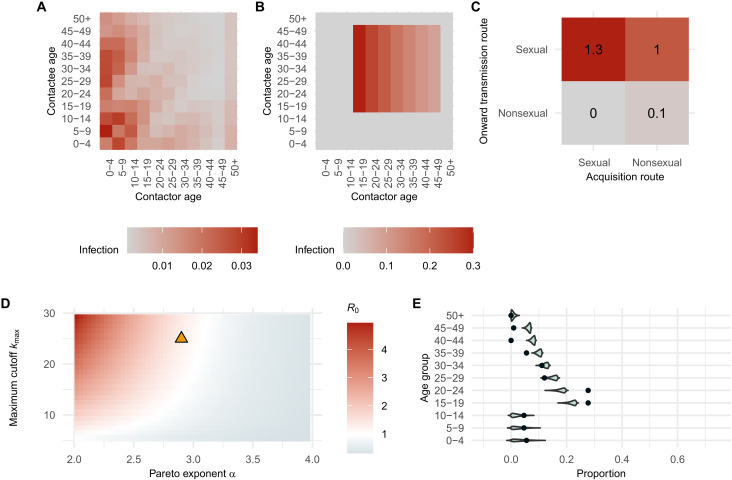
Projected next-generation matrices, reproduction numbers, and age distribution of infection. (**A**) Submatrix of the NGM representing transmission via nonsexual contacts from cases that were infected via nonsexual contact. (**B**) Submatrix of the NGM representing transmission via sexual contacts from cases aged 15 years and older who were infected via sexual contact. (**C**) Summarized route-to-route reproduction numbers. (**D**) Projected range of basic reproduction numbers as a function of the Pareto exponent (lower values indicate heavier-tailed degree distributions) and the maximum cutoff (maximum number of sexual partners). Yellow triangle indicates the baseline scenario. (**E**) Projected age distribution of infection incidence (blue density plots) compared to observed case data (black dots) from Kamituga, South Kivu province, the Democratic Republic of the Congo, between October 2023 and March 2024.

The submatrix of the NGM for transmission from-nonsexual-to-nonsexual contacts projects higher secondary infections among young children or from children to older adults in their 20s and 30s (Fig. 1A). Secondary infections via transmission from-sexual-to-sexual contacts were mainly produced by younger adults aged 15 to 19 (Fig. 1B), using a baseline scenario with parameterization of the model for Kamituga (see the details in Materials and Methods and the Supplementary Materials). We can quantify the relative contribution of transmissions within and between the two routes by projecting reproduction numbers for each submatrix under the baseline scenario (Fig. 1C). To determine the overall transmission potential $R_{0}$ (the basic reproduction number, where $R_{0}>1$ indicates that an outbreak will grow), the full NGM is required to capture the interaction within and between transmission routes (see the proof in the Supplementary Materials).

In addition to the baseline scenario, we accounted for the presumed uncertainty in the current estimates of parameters concerning the infectious period and SARs for clade Ib MPXV, and parameters around sexual behavior. We projected route-to-route–specific reproduction numbers across the possible parameter range using Latin hypercube sampling (*38*) (see Materials and Methods for details on the explored parameter spaces). The projected values varied by route of transmission, ranging from 0.12 to 1.45 for $R_{s,s}$, from 0.28 to 1.11 for $R_{s,\text{ns}}$, from 0 to 0.07 for $R_{\text{ns},s}$, and from 0 to 0.25 for $R_{\text{ns},\text{ns}}$. The projected basic reproduction number, $R_{0}$, was 1.31 for the baseline scenario and ranged from 0.15 to 1.48. The basic reproduction number $R_{0}$ was primarily determined by $R_{s,s}$ and $R_{s,\text{ns}}$, the potentials for from-sexual-to-sexual and from-nonsexual-to-sexual transmission, respectively. $R_{0}$ was sensitive to the change in the assumed distribution of the number of sexual partners (degree) (Fig. 1D). $R_{s,s}$ and $R_{s,\text{ns}}$ responded in a qualitatively similar manner to changes in the assumed degree distribution (fig. S1). A higher maximum number of partners an individual can have at a time and a heavier-tailed degree distribution led to an increased overall transmission potential $R_{0}$. Our derivation of the NGM elements also suggested that the expected number of secondary infections caused by transmission via sexual contact is determined by the first and second moments of the degree distribution (see the Supplementary Materials). This means that we can project the transmission potentials accurately if the mean and variance of the degree distribution are available, without knowing the entire degree distribution.

We projected a normalized age distribution of incidence of infection during an exponential growth phase by using the NGM and compared it with the observed age distribution of clade Ib cases in Kamituga between October 2023 and March 2024 (*1*) (see Materials and Methods for details on the computational procedure). The projected age distribution closely aligned with the observed data, despite the wide range of parameter sets explored, indicating empirical support for the model structure used in this study (Fig. 1E). The relative incidence among late teens and early 20s was higher in the observed data, likely owing to how the actual outbreak started with cases that belonged to those age groups in Kamituga.

### Time-varying contribution to transmission dynamics by route of transmission and age group

**Fig. 2.**
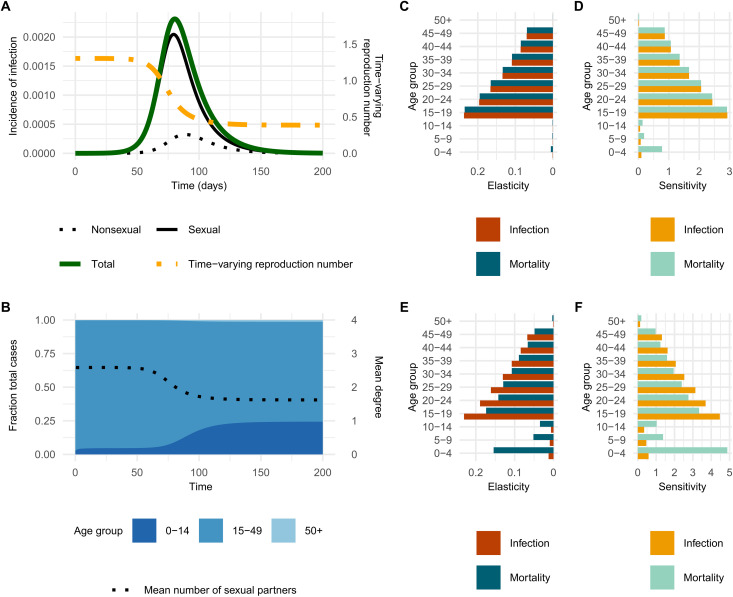
Projected epidemic dynamics over time by transmission route and age group. (**A**) Projected incidence of infections per day by transmission route shown alongside the corresponding time-varying reproduction number. (**B**) Age distribution of incidence of infection over time, shown alongside the mean number of sexual partners among newly infected individuals (black dotted line). (**C** and **D**) Elasticity and sensitivity of the next-generation matrix (NGM) and mortality-weighted NGM at the early stages of the epidemic. (**E** and **F**) Elasticity and sensitivity metrics at a later stage (day 200) of the epidemic.

We projected the time-varying contribution of each route of transmission and age group, both by simulating an epidemic and by analytically deriving the time-varying NGM of the model (see the Supplementary Materials). First, the projected epidemic trajectories under the baseline scenario are presented in Fig. 2A. This result shows that transmission via sexual contacts dominates the dynamics in the initial phase of an epidemic in terms of higher relative incidence compared with transmission via nonsexual contacts. Later in the epidemic, the relative share of transmission via nonsexual contacts becomes more prominent, leading to higher total incidence of infection. In this scenario, the time-varying reproduction number R(t) (computed as the dominant eigenvalue of the time-varying NGM, see the Supplementary Materials) was 1.3 at the beginning (the projected $R_{0}$) and started declining as transmission via sexual contacts becomes less dominant. In the later phase, R(t) declined to an asymptote of 0.45 due to the accumulated immunity among sexually active groups, leading to a self-contained outbreak. We performed a sensitivity analysis with the community contact matrix that includes all nonhousehold contacts. This analysis showed that the shift in transmission from sexual to nonsexual contacts occurred earlier due to the higher transmission rate via nonsexual contacts (fig. S2).

In real-world outbreaks, the underlying dynamics cannot be directly observed. The network epidemic model offers two ways of assessing the epidemic phase over time using observable epidemiological data: the time-varying age distribution of relative incidence and the time-varying mean degree of cases (Fig. 2B). The age distribution of relative incidence is concentrated among sexually active age groups when transmission via sexual contacts dominates the incidence. As its contribution declines over the course of the epidemic, the relative incidence increases among the younger, sexually inactive age groups, even when the total incidence is decreasing. The declining contribution of transmission via sexual contacts can also be measured by monitoring the degree of cases. In the baseline projection, the mean degree of newly infected sexually active individuals starts at 2.6 at the beginning and rapidly declines to an asymptote of 1.6. This shows that individuals infected early in the epidemic typically have a higher number of partners than those infected later, when transmission via sexual contacts contribute less to the dynamics.

We use targeted vaccination as an example to identify subgroups with the highest expected contribution to two epidemic outcomes: the number of secondary infections after one generation of transmission (i.e., the reproduction number) and overall mortality. These contributions were measured in terms of cumulative sensitivity and elasticity. Sensitivity reflects the absolute change in an outcome resulting from a unit change in a parameter. Elasticity represents the proportional change in the outcome resulting from a proportional change in that parameter. These metrics quantify the relative and absolute contributions of each age group to infection and mortality. The analysis is based on perturbing the (time-varying) NGM and mortality-weighted NGM by immunizing one susceptible individual in a given group, as would occur through vaccination (see Materials and Methods and the Supplementary Materials). We present the quantified age group– and outcome-specific contributions at the initial stages of an epidemic (Fig. 2, C and D) and on day 200 (Fig. 2, E and F). In the initial stages of the epidemic, younger adults aged 15 to 24 years contribute to both epidemic outcomes the most, making them the optimal target group for interventions. However, on day 200, the relative contribution to onward transmission declined among sexually active age groups, and the groups contributing to the overall mortality most were young adults aged 15 to 19 years and children aged 0 to 4 years (Fig. 2, E and F). Consequently, on day 200 after the initial outbreak, targeting both younger adults (15 to 19 years) and younger children (0 to 4 years) would be the most effective to minimize the expected overall mortality. We found that higher relative contributions of 0 to 4 years to the overall mortality would arise around day 160 to 200 by computing the time-varying age-specific sensitivity and elasticity over time (fig. S3). We performed a sensitivity analysis using an alternative mortality-weighting approach based on crude CFRs for clade Ib in South Kivu (*3*). The relative contribution of the 0 to 4 year age group to overall mortality was diminished compared to the main analysis, due to the substantially smaller differences in age-specific CFRs used in the sensitivity analysis (fig. S4).

### Effect of transmission route on epidemic size

To examine how increased transmissibility via sexual and nonsexual contacts affects the epidemic size (fraction of the population that is ultimately infected if no interventions were taken), we projected epidemic trajectories and cumulative infections under different parameter scenarios by varying SARs for each transmission route (Table 1). Four scenarios were explored by lowering SAR via sexual contacts and increasing SAR within households (household SAR), while keeping the same $R_{0}=1.31$ as the baseline. With the baseline scenario, the epidemic size was the smallest (9.1%), and transmission via sexual contacts consisted of 84% of the total infections. In scenarios with higher household SAR, the epidemic size increased, driven by the increased proportion of transmission via nonsexual contacts interacting with transmission via sexual contacts ($R_{\text{ns},s}$). A moderate increase in the value for nonsexual-to-nonsexual transmission of $R_{\text{ns},\text{ns}}$ from 0.11 to 0.67 was sufficient to shift the dominant route of transmission from sexual to nonsexual contacts. This resulted in a decrease from 89 to 41% of total infections caused by transmission via sexual contacts (comparing baseline to scenario B). These “switching” dynamics align with a theoretical prediction from a previous study (*23*). This finding is relevant because this moderate value for nonsexual-to-nonsexual transmission of $R_{\text{ns},\text{ns}}$ around 0.4 to 0.6 overlaps with the historical estimates of reproduction numbers for clade Ia MPXV (*39*, *40*). The assumed household SAR used to determine $R_{\text{ns},\text{ns}}$ was also comparable to the values reported in previous (non–clade Ib) mpox outbreaks (*41*). Moreover, the interaction between the two routes of transmission is crucial in the epidemic outbreak. In scenario C, where the reproduction number of each of the four submatrices of the NGM are below 1, an outbreak can still occur ($R_{0}=1.31$), leading to a larger epidemic size than in the baseline scenario. In all alternative scenarios, larger epidemic sizes were projected with greater household SAR, even though the values of $R_{\text{ns},\text{ns}}$ were smaller than or closer to 1. Larger epidemic sizes were driven by the earlier shift in the dominant relative incidence from transmission via sexual contacts to transmission via nonsexual contacts (see the projected epidemic curves for each scenario in fig. S5).

**Table 1.** Epidemic size and contribution of sexual transmission under varying SARs. Epidemic size and percentage contribution of transmission via sexual contacts to the total epidemic size are shown across scenarios with different assumed SARs for transmission via sexual (s) and nonsexual (ns) contacts, while maintaining a constant basic reproduction number ($R_{0}=1.31$). In scenario C, the household SAR is set to 0.49 (instead of 0.50) to ensure that all reproduction numbers remain below 1.

Scenario	Sexual SAR*	Household SAR*	$R_{0}$ *	$R_{s,s}$	$R_{\text{ns},\text{ns}}$	$R_{s,\text{ns}}$	$R_{\text{ns},s}$	Epidemic size (%)	Contribution of transmission via sexual contacts (%)
Baseline	0.80	0.10	1.31	1.28	0.11	0.95	0.03	9.1	89
A	0.74	0.30	1.31	1.18	0.43	0.88	0.12	28	54
B	0.67	0.40	1.31	1.07	0.67	0.79	0.19	46	41
C	0.50	0.49	1.31	0.80	0.97	0.59	0.28	62	32

* Secondary attack risks (SARs) are used as inputs to derive per-contact transmission probabilities. $R_{0}$ and the four reproduction numbers are computed from the corresponding transmission probabilities.

## DISCUSSION

Our results suggest that distinct dynamics of clade Ib mpox transmission may be driven by the coexistence of two transmission routes. The dominant route of transmission may shift over time from transmission via sexual contacts to nonsexual contacts, depending on their relative transmission potentials. Such shifts may be detected using observable proxies, such as changes in the age distribution of cases or in the mean number of sexual partners among confirmed cases, when measured consistently over time. If transmission via sexual contacts swiftly declines but transmission via nonsexual contacts continues over a prolonged period, that may indicate a higher transmission potential via close physical contacts in households or communities, leading to larger epidemic sizes. Monitoring such switching dynamics is crucial for anticipating the potential outcome of long-term epidemics and for informing response strategies in regions that may face future introductions of clade Ib MPXV.

Optimal target groups for intervention may differ, depending on both the epidemic outcome to be minimized and the timing of intervention. In our projections, secondary infections via nonsexual contacts were primarily associated with young children, whereas those via sexual contacts were concentrated among younger adults. Elasticity analyses indicated that targeting sexually active age groups, especially those aged 15 to 24 years, would be the most effective intervention to reduce both infections and mortality in the initial phase of an epidemic. However, once an epidemic progresses and immunity accumulates, the effectiveness of such intervention strategies on sexually active age groups would be attenuated. If increasing incidence rates among young children are observed, this may imply that sexually active groups may no longer be the optimal target groups for intervention. In contrast, those groups may remain relevant for outbreak responses in countries or regions that are still in their initial phase or have not yet experienced mpox outbreaks. These considerations are important for planning response strategies for ongoing and potential outbreaks, where anticipating the risk of sustained outbreaks following importation events is essential.

Our analyses underscore two critical data gaps: the SARs for each transmission route, and the sexual partnership distribution over relevant timeframes, particularly from the affected settings. Our analyses refer to the range of household SAR estimates reported from historical outbreaks (*41*), but these values likely represent an upper bound for community transmission rates and may not reflect the characteristics of the current outbreaks. Sexual behavior data are often reported on annual scales (*42*), which may not reflect the sexual contacts during the much shorter timescales for the typical infectious period of MPXV (*43*, *44*). The simulated ranges of the reproduction number $R_{0}$, the potential for sexual-to-sexual transmission ($R_{\text{ss}}$), and nonsexual-to-sexual transmission $\left(R_{s,\text{ns}}\right)$ under varying degree distributions for the number of sexual partners highlighted that the misspecification of the degree distribution can lead to incorrect estimates of reproduction numbers. Modest increases in SARs for transmission via nonsexual contacts could also be sufficient to shift the dominant transmission route and increase the epidemic size. These insights are essential to anticipate further international spread of clade Ib MPXV beyond the currently affected population groups and countries.

There are several limitations to this study. First, there are simplifying assumptions in our model. We assumed that both transmission routes had the same underlying generation time, although recent studies reported that the realized generation time was shorter for transmission via sexual contacts than for transmission via nonsexual contacts (*8*). Our model structure is based on a closed population and does not incorporate spatial transmission or importations of cases. It also does not account for sex-specific differences, despite evidence of early involvement of female sex workers in Kamituga (*1*, *11*), differing age-sex distributions in sexual contacts (*45*), and age- and sex-dependent sexual mixing (*46*). Implementing these features would require additional demographic and sexual network data and a more complex model structure beyond the configuration network approach used here (*26*). Adding further age assortativity would likely lead to more strongly confined transmission within sexually active age groups and propagation to sexually inactive age groups may occur more slowly. These additions would not negate our main conclusion regarding the shift in dominant transmission route. Second, contact matrices and the degree distribution used in this study may not reflect the actual contact patterns during the outbreak in Kamituga, for instance, due to a precautionary response as seen in the 2022 global outbreak (*47*). The actual sexual contact network may be partly dynamic, and one-time sexual contacts, such as transactional sex, are not explicitly modeled and assumed to be represented by higher degrees of the partnership distribution. Empirical data are needed to determine the sexual partnership distribution, the age at the start of sexual activity (*48*), and the sex and age dependency of sexual behaviors when applying the proposed framework. Third, our analysis does not account for preexisting or heterogeneous susceptibility in the population, such as partial immunity among older adults due to smallpox vaccinations (*49*). The proposed approach could be further refined by incorporating conditions such as nutritional status and immunity (*45*), which may affect susceptibility and disease severity in target groups. Fourth, although we illustrated the impact of age-specific targeted interventions, our analytical framework could be extended to degree-specific interventions. Identifying individual partnership degrees is challenging, however, and such analyses would require nonproportional perturbation of NGMs. Last, the quantities presented in this study should not be interpreted as empirical estimates, but rather as model-projected values, conditional on assumed scenarios. We focus on projecting potential epidemic outcomes under a range of plausible conditions, rather than reconstructing the specific dynamics in Kamituga or inferring epidemic characteristics such as the duration of the outbreak, which is more prolonged in the actual outbreak. While model calibration to case data from Kamituga is theoretically feasible, such inferences would require careful interpretation, given the limitations imposed by armed conflict and limited surveillance infrastructure (*50*).

Our model can be applied to support risk assessments of ongoing mpox outbreaks, including in Kinshasa where clades Ia and Ib are cocirculating (*51*) and the new clade IIb outbreak in Sierra Leone (*16*). In both places, adults aged 16 to 35 years have been the most affected age groups. This study also contributes to the advance in modeling emerging infections with multiple, interacting transmission routes within age-structured populations with a network structure. Our mathematical analysis and projected epidemic sizes demonstrate that interactions between different transmission routes play a crucial role in shaping transmission dynamics, and that discarding them may lead to completely different epidemic outcomes. The proposed approach links qualitative understanding about transmission dynamics with empirical data and provides a simple principle for identifying optimal subgroups for targeted interventions given available observations.

In conclusion, our findings highlight the critical need to adapt intervention strategies to the evolving dynamics of mpox outbreaks. In the presence of multiple transmission routes, models that explicitly capture their coexistence and interaction, such as the one proposed here, are essential for anticipating future epidemic trajectories. Optimal control strategies vary over time, depending not only on the epidemic phase but also on the specific outcome to be minimized, whether it be total infection or mortality. For countries that are at risk for importing new mpox cases, this highlights that continuous monitoring remains crucial to detect shifts in the dominant route of transmission and to support timely, effective public health responses.

## MATERIALS AND METHODS

### Two-level network epidemic model

The network epidemic model can be described by a set of ODEs. We first outline how contacts are represented in the model and then how transmission occurs through these contacts. There are two types of contacts in the model: sexual and nonsexual. Sexual contacts are modeled using a configuration network construction with a prespecified degree distribution (*35*), where the degree denotes the number of sexual partners per individual over the time window of a typical infectious period, under the assumption that partnerships remain fixed over the course of the epidemic. Nonsexual contacts occur across the entire population for all individuals through age-dependent random mixing, represented by a contact matrix whose elements denote the average number of daily contacts per individual between and within age groups.

The two types of contacts are linked through individuals who participate in both. Transmission follows a compartmental SEIR process and can occur via either sexual or nonsexual contacts, depending on the route of contact between an infectious and a susceptible individual. The transmission probability per sexual partnership is the same and independent of the total number of sexual partners of the individuals in the partnership. Our model is deterministic, using ODEs to describe the transmission dynamics across different sexual partnership degrees and age groups, rather than simulating stochastic events (*32*). The model is fully parametrized by three sets of parameters: (i) degree distribution relating to sexual contacts, (ii) contact matrix relating to nonsexual contacts, and (iii) infection parameters. Moreover, we focus on projections of transmission potential and epidemic trajectories over a period of less than 1 year; we ignore aging or demographic turnover (e.g., migration, births, and natural deaths) as well as the waning and the preexistence of immunity. Full model details are provided in the Supplementary Materials.

### Next-generation matrices and reproduction numbers

To quantify the age-specific contribution to transmission, we first introduce the NGM K, which contains as its elements the number of secondary infections by age group and transmission route. By summarizing all elements, the full NGM K can be expressed as

$$K=\left(\begin{array}{c} K_{s,s}\;K_{s,\text{ns}}\\ K_{\text{ns},s}K_{\text{ns},\text{ns}} \end{array}\right)$$

where the four block submatrices can be viewed as the NGMs that describe the secondary transmission from one route to another or within the same route in the absence of other route-to-route transmissions: $K_{s,s}$ (transmission from sexual to sexual contacts), $K_{s,\text{ns}}$ (from nonsexual to sexual), $K_{\text{ns},s}$ (from sexual to nonsexual), and $K_{\text{ns},\text{ns}}$ (from nonsexual to nonsexual). Each submatrix is an M × M matrix with M being the number of age groups and consists of the elements of the age-specific number of secondary infections caused via one route of transmission by a case infected via a specific route of transmission. The four submatrices are necessary as there is dependence on the transmission route through which the index case got infected. We derive all NGMs from the network epidemic model developed above, and detailed derivations and their interpretations are provided in the Supplementary Materials.

We then characterize the route-to-route–specific transmission potential and the overall dynamics by computing distinctive reproduction numbers. The dominant eigenvalue of the full NGM at time 0 (i.e., the time at which all individuals are susceptible) is the basic reproduction number $R_{0}$, determining whether a major outbreak may occur or not (*32*, *37*). Route-specific reproduction numbers can be computed by taking the dominant eigenvalue of the four submatrices defined above, and therefore we can obtain four quantities: $R_{s,s}$ (from sexual to sexual), $R_{s,\text{ns}}$ (from nonsexual to sexual), $R_{\text{ns},s}$ (from sexual to nonsexual), and $R_{\text{ns},\text{ns}}$ (from nonsexual to nonsexual). Note that these reproduction numbers are derived with an implicit assumption that there are no interactions between transmission routes due to the block-wise decoupling of the full NGM. Therefore, they should be interpreted as the average number of secondary infections within each route pair with an assumption that no cross-transmission occurs between routes. To determine $R_{0},$ the full NGM K is needed. By further extending this next-generation approach, one can derive time-varying NGMs and compute the time-varying reproduction number R(t) (Supplementary Materials) and use R(t) to analyze the evolving overall transmission potential.

### Perturbation analysis of next-generation matrices

By analyzing the constructed NGMs at different points in time, we can determine the age group that contributes most to onward transmission at each time point. Such age-specific contributions can be measured by two indicators: cumulative sensitivity ${\bar{s}}_{m}$ and cumulative elasticity ${\bar{e}}_{m}$ (for age group m). Sensitivity measures the absolute change (hereafter R, dropping t for simplifying notation) with respect to a small change in an element of an NGM (i.e., $s_{\mathrm{ij}}=\frac{\partial R}{\partial k_{\mathrm{ij}}}$). Elasticity measures the proportional (relative) change in response to a proportional (relative) change in an element of an NGM and is expressed by $e_{\mathrm{ij}}=\frac{\partial R}{\partial k_{\mathrm{ij}}}\frac{k_{\mathrm{ij}}}{R}$. Cumulative sensitivity and elasticity quantify the total impact of a small (absolute and relative) change in a single targeted age group i on R, and they are given by ${\bar{s}}_{i}=\sum_{j}s_{\mathrm{ij}}$ and ${\bar{e}}_{i}=\sum_{j}e_{\mathrm{ij}}$, respectively (*32*, *52*).

The perturbation analysis described above can be applied to other projection matrices (*33*, *53*). We constructed a projection matrix for death due to secondary infections at time t by multiplying a matrix of age-specific infection mortality ratio

$$D_{t}=MK_{t}$$

where M is a diagonal matrix with the elements of age-specific infection mortality ratios, and $K_{t}$ is the (full) NGM at time t.By quantifying age-specific cumulative sensitivities or elasticities, we can determine the group that contributes most to the mortality due to secondary infections caused by that group. This approach is extendable to other disease outcomes or indicators (e.g., hospitalizations or disability-adjusted life years) as long as the outcome can be expressed as a multiplicative effect, as demonstrated in (*33*). For the infection mortality matrix M, we used the pooled CFR estimates of clade I reported by Whittles *et al.* (*10*) as a proxy in the main analysis. As a sensitivity analysis, we also used the crude CFR values for South Kivu reported by WHO (*3*).

### Parameterization of contact networks

In our network epidemic model, age-specific nonsexual contacts were parameterized by using synthetic contact matrices estimated for the DRC by Prem *et al.* (*36*). Contacts in the contact matrices are defined as conversational and/or physical contacts as used by social contact surveys (*54*, *55*). Contacts in the household matrix are assumed to largely represent individuals’ interactions with close physical proximity. We therefore used the household contact matrix for the main analysis and the contact matrix that covers contacts in all settings (i.e., contacts within and outside the household, including contacts in a community) for the sensitivity analysis (fig. S6). While the contact matrices do not distinguish contact types by conversational or physical contact, it is likely that nonhousehold settings include a higher proportion of nonphysical (i.e., conversational-only) contacts. Therefore, the projections in the sensitivity analysis should be interpreted as upper-bound estimates under this assumption as our use of the all-settings matrix may overestimate the contact rates for transmission via nonsexual contacts.

To represent the sexual contact network, we used a degree distribution that represents the number of sexual partners in the time window of a typical infectious period. We used a truncated power-law degree distribution given by

$$p_{k}=\left\{ \begin{array}{cc} N_{0} & \text{for}\;k=0\\ Ck^{-\alpha } & \text{for}\;1\leq k\leq k_{\text{max}}\\ 0 & \text{for}\;k>k_{\text{max}} \end{array}\right.$$

where $N_{0}$ is the proportion of individuals with zero partners, α is the Pareto exponent, C is a normalization constant, and $k_{\text{max}}$ is the maximum degree. The same formulation is applied in, e.g., (*23*). The truncated power-law distribution allows for empirical parameterization of the proportion of degree zero, as well as the maximum cutoff. As a baseline parameter set to visualize the main results, we used $N_{0}=0.14$ based on a Demographic and Health Survey in the DRC (*45*), α=2.9 by referring to an estimated value for men in a sexual behavior survey in Burkina Faso (Burkina Faso permits legal polygamy and has documented the presence of transactional sex work, similar to the DRC) (*56*), $k_{\text{max}}=25$ to reflect the mean number of clients per female sex worker over an infectious period of 10 days by linearly rescaling the observed number of clients over a week (*57*). Although the baseline parameter values and the consequent mean number were similar to the observed data of the number of sexual partners (*12*), there are large uncertainties in each parameter. Moreover, a broad range of parameter values could also be used for assessing how the outbreaks may develop in other countries that have different epidemiological and behavioral parameters than those of the DRC. Therefore, we took a range of each parameter, rather than using point estimates, when comparing the model projection to the observed age distribution of incidence of infection using a Latin hypercube sampling (*38*) (see the summary of explored parameter ranges in table S1). We assumed the same degree distribution for all sexually active age groups [those aged 15 to 49 years, based on (*45*)]. Age groups were defined as 0 to 4, 5 to 9, 10 to 14, …, 40 to 44, 45 to 49, and 50+ years old, according to the age grouping in WHO reports (*3*).

### Epidemiological parameters

For the baseline scenario, we used a mean generation time of 10 days [based on the mean serial interval estimates for clade IIb cases in 2022 (*58*) and clade Ib cases infected through nonsexual contacts in eastern DRC (*8*)], together with a mean latent period of 2 days (*59*), and a mean infectious period of 8 days (*60*). We opted for this parameter choice to better reflect the outbreaks in Kamituga, rather than relying on pooled estimates from recent systematic reviews (*61*, *62*). Per-contact transmission rates for transmission via sexual and nonsexual contacts were specified by referring to the SAR observed in historical clade Ia outbreaks and clade IIb outbreaks, and a household SAR of 0.10 and a sexual SAR of 0.80 were used for the baseline scenario. We assumed the same per-contact transmission rates for both within-household and outside-household contacts as the focus of the sensitivity analysis of fig. S2 was to check the impact of differential contact rates. Because both the natural history of clade Ib MPXV is still largely unknown, and to assess how outbreaks may develop in other settings, we explored the wide range of the parameter space to project reproduction numbers and age distribution of incidence of infection. The explored range of the transmission parameters are summarized in table S1, and its rationale is described in the Supplementary Materials.

When computing epidemic sizes under different scenarios (Table 1), the SAR of household transmission is varied, while keeping the basic reproduction number fixed to the baseline setting $R_{0}=1.31$. Varying the SAR for household transmission fully determines the corresponding transmission parameter. The parameter of transmission via sexual contacts is determined through the basic reproduction number. As a consequence, the SAR of transmission via sexual contacts is modified as well. Scenario C with a SAR for household transmission of 0.49 instead of 0.50 is chosen to ensure that $R_{\text{ns},\text{ns}}<1$.

### Projected age distribution of incidence of infection

The projected age distribution of incidence of infection was computed by taking the dominant (right) eigenvector of the NGM, based on its ergodic property (*32*, *53*). The dominant right eigenvector converges to a stable distribution, which is proportional to the incidence of infection during the exponential growth phase. We summed the secondary infections from both transmission routes (sexual and nonsexual), computed the resulting age-specific incidence, and then normalized it to obtain the projected age distribution of incidence. To assess convergence, we evaluated the damping ratio, defined as the ratio of the largest to the second-largest eigenvalue (*53*), indicating that the asymptotic distribution is reliably reached (with an error smaller than 1% of the dominant eigenvalue) after three generations of transmission.

### Outbreak data and demographic information

We collected the number of confirmed mpox cases in Kamituga, South Kivu province, the DRC, between October 2023 and March 2024 (*1*). We aggregated sex to construct a (normalized) age distribution of incidence of infection. The nationwide population age distribution in the DRC was retrieved from (*63*), assuming that the distribution is comparable in Kamituga to compare the observed incidence against the model-projected incidence.

## Supplementary Materials

### This PDF file includes:

Supplementary Text Figs. S1 to S6 Table S1 References
