# Supplementary material for: Dynamic shift in the dominant transmission route of clade Ib monkeypox virus across networks with sexual and nonsexual contacts
Source: Sci Adv. 2026 Apr 1;12(14):eaec1931. doi: 10.1126/sciadv.aec1931 (PMC13041746; doi:10.1126/sciadv.aec1931)
Supplement: Supplementary file 1 — Supplementary Text Figs. S1 to S6 Table S1 References [file sciadv.aec1931_sm.pdf]

Science Advances
AAAS

Supplementary Materials for

Dynamic shift in the dominant transmission route of clade Ib monkeypox virus across networks with sexual and nonsexual contacts

Fuminari Miura *et al.*

Corresponding author: Fuminari Miura, fuminari.miura@rivm.nl; Ka Yin Leung, k.leung@scp.nl

Sci. Adv. **12**, eaec1931 (2026)
DOI: 10.1126/sciadv.aec1931

This PDF file includes:

Supplementary Text
Figs. S1 to S6
Table S1
References

Supplementary Text

Supplementary text 1: Additional detail on parameter settings, model assumptions and scenarios considered

Explored ranges of epidemiological parameters

The parameter ranges were specified by referring to historically observed values: mean generation time of 7–17 days, mean latent period of 0–2 days, and mean infectious period of 7–17 days. The range of mean generation time reflected the shortest scenario referring to clade IIb MPXV outbreaks in 2022 (*58*) and the longest scenario based on historical estimates for clade Ia (*64*). The shortest mean latent period was set as 0 days to represent possible infectiousness via sexual contact before experiencing recognizable symptoms. We referred to the reported range of household secondary attack risks (from 0 to 12%: 0-11% among countries in Africa (*41*), 12% in a historical outbreak in the DRC (*65*), and 7% in the US during the 2022 outbreak (*15*)).

Translating secondary attack risks into transmission rates

As the secondary attack risk per sexual partner can be computed as 1 minus the probability of escaping from all infectious contacts from a single sexual partner over the infectious period, the relationship between transmission rate β and the SAR of transmission via sexual contact ϕ_S is given by $\phi_S = 1 - \int_0^\infty exp(-\beta t) f_\tau(t) dt$ where τ is the infectious period with density function f_τ. In our SEIR framework, $f_\tau(t) = \gamma \exp(-\gamma t)$, so $\phi_s = \frac{\mathrm{b}}{\mathrm{b}+\gamma}$. By rearranging this, the transmission rate for sexual contacts is given by $\mathrm{b} = \frac{\phi_S}{1-\phi_S}\gamma$. An implicit assumption here is that the number of contacts per partner per unit time is 1 (or, the contact frequency per day is involved in the transmission rate b). For the transmission rate in households or communities, we denote b_H by a household transmission rate, and ϕ_H the SAR of transmission within households. The household transmission rate satisfies $\mathrm{b}_H = p\overline{\mathrm{c}_w}$ where $\overline{\mathrm{c}_w}$ represents the average effective contacts per day within a household and p is the per-contact probability of infection. $\overline{\mathrm{c}_w}$ is computed by taking the dominant eigenvalue of age-weighted contact matrix $\mathrm{C}_w = [\mathrm{c}_{ij}\pi_j]$, i.e., $\overline{\mathrm{c}_w} = \rho(\mathrm{C}_w)$. Using the same relationship between SAR and transmission rate as above, we obtain $p = \frac{\phi_H}{1-\phi_H}\frac{\gamma}{\overline{\mathrm{c}_w}}$. For the baseline scenario, the resulting per-contact probability of infection for non-sexual contacts is 0.025.

Additional model assumptions

In addition to the structural assumptions of the network model, we outline below several simplifying assumptions made when illustrating our modeling framework:

- No differences in sex in the model: we assume that contact rates (both sexual and non-sexual contact rates), the age distribution, and any other model parameters are not sex specific.
- Equal per-contact susceptibility and infectivity: we assume homogeneous susceptibility and infectivity across individuals, regardless of age and sex, due to limited data. That is, all individuals are equally likely to acquire and transmit infection given contact.
- No prior immunity existing in the population: the model assumes a fully susceptible population at the onset of the outbreak, reflecting the absence of prior immunity or protective effect from historical smallpox vaccination, which ended around 1980 in the DRC (*49*). We opted for this conservative assumption given the limited information on cross-protection, waning immunity, and vaccination coverage in the affected populations.
- Vaccine efficacy and vaccine mode of action: when simulating targeted interventions with the sensitivity of NGMs, we consider the decrease of susceptible proportions among the targeted age groups. We assume that the vaccine confers protection against infection through

an all-or-nothing mechanism (i.e., a proportion of vaccinated individuals are assumed to be fully protected from infection, while the remainder receives no protection). Vaccine efficacy (VE) is defined as the proportion of vaccinated individuals who are completely protected against infection, and VE against infection assumed to be 0.8 (*66*, *67*).

- Initial conditions of numerical simulations: the model assumed that the epidemic is seeded by infectives in the Exposed stage (infected but not yet infectious) in the sexually active age group 20-24 years old who form a fraction 1/population size (i.e., 9×10^{-6}) of the total population, which has a small effect at the very beginning of the epidemic only.

Supplementary text 2: Mathematical details

1 Model description

We consider a population that is structured according to age and sexual activity. Whether an individual belongs to the sexually active population or not depends on age (younger age groups are assumed to be sexually inactive). An individual who is part of the sexually active population has a certain (fixed) number of sexual partners n that is called the degree of the individual. The degree of an individual is determined by an age-*independent* degree distribution $(p_k)_{k=0}^{\infty}$, where p_k is the fraction of the population with degree k, k=0,1,2,… The network does not change in the period under consideration. A proportion of the sexually active population can be without partners, i.e. individuals with degree 0, where $p_0 > 0$.

There are two types of contacts that can be made in the population. Individuals in the sexually active population can make (sexual) contact within their partnerships. On top of that, there are non-sexual contacts between any two individuals in the population according to the mass action principle. These non-sexual contacts are age-*dependent*. The sexual network is constructed by the configuration network construction (*35*). We follow notation and terminology of (*31*). In the configuration network construction, an individual with degree n is considered to be a collection of n binding sites that are conditionally independent (also referred to as "half-edges" or "stubs" in graph theory). This mathematical convenient perspective facilitates the formulation and analysis of this class of models: probabilities defined at the binding site level determine individual-level probabilities, which in turn enable the analysis of the population-level dynamics (*26*, *31*).

We consider a SEIR infection in the population with the sexual network and non-sexual contacts. Individuals are either Susceptible, Exposed to infection but not yet infectious, Infectious, or Recovered and immune from the infection for the duration of the epidemic. The latent period is exponentially distributed with mean $1/\sigma$, and the infectious period is exponentially distributed with mean $1/\gamma$. Latent and infectious period are independent of the route of transmission or the ages of the individuals involved. Transmission via sexual contact occurs at rate β from an infectious individual to each of his/her susceptible sexual partners, independently of the total number of partners. Transmission via non-sexual contact occurs at rate pc_{ij} from an infectious individual of age i to a susceptible individual of age j, where $c_{ij} = c_{ji}$ is the per-pair non-sexual contact rate between individuals of age i and j, and p is the probability of transmission upon non-sexual contact between an infectious and susceptible individual.

The configuration network construction allows us to determine the state of an individual by considering the age and infection status of the individual. If the individual is sexually active, the state of an individual involves also the number of partners of the individual, and the age and infection status of each of these partners. Because sexually active individuals can be viewed as collections of binding sites, we can reduce the description of the model to tracking susceptible binding sites, together with susceptible, exposed, and infectious individuals in the sexually inactive ages. This enables the analysis of the model.

We let $(\pi_m)_{m=1}^{M}$ denote the population age distribution, where π_m is the proportion of individuals in age group m. The age groups $m = 1, \dots, N_1 - 1, N_2 + 1, \dots, M$ comprise the sexually inactive population, and the age groups $N_1, \dots, N_2$ form the sexually active population, $1 \leq N_1 < N_2 \leq M$. Consider a sexually active susceptible individual (i.e., age group $N_1 \leq i \leq N_2$) with degree $n > 0$, and examine one of his/her binding sites. Assume that the individual does not become infected

through one of his/her $n-1$ other binding sites for the period under consideration. Let $x_{(i,n),(j,y)}$ denote the probability that a binding site belongs to a susceptible individual with age i and degree n and is occupied by an individual with age j and infection state y, $y = S, E, I, R$. Let $x_{i,0}$ denote the probability an individual with age i and degree 0 is susceptible. Let $\chi_{(i,n),(j,k,S)}$ denote the probability that a binding site belongs to a susceptible individual with age i and degree n and is occupied by a susceptible individual with age j and degree k. Note that consistency requires $x_{(i,n),(j,S)} = \sum_{k=1}^{\infty} \chi_{(i,n),(j,k,S)}$.

For each age group $1 \le i \le M$, let S_i, E_i, I_i, R_i denote the fraction of the population that has age i and infection state S, E, I, or R. Note that $S_i + E_i + I_i + R_i = 1$. Note that a *sexually active individual* is of age j with probability

$$\tilde{\pi}_j = \frac{\pi_j}{\sum_{l=N_1}^{N_2} \pi_l}. \qquad (1)$$

For convenience of notation, we let the vector $\boldsymbol{\pi}_{\mathbf{S}}$ be

$$\boldsymbol{\pi}_{\mathbf{S}} = \left(0, \dots, 0, \tilde{\pi}_{N_1}, \dots, \tilde{\pi}_{N_2}, 0, \dots, 0\right)^T.$$

The size-biased degree distribution is

$$\tilde{p}_n = \frac{n p_n}{\sum_{k=1}^{\infty} k\, p_k}. \qquad (2)$$

A key variable is

$$\bar{x}_{(i,n),j} = x_{(i,n),(j,S)} + x_{(i,n),(j,E)} + x_{(i,n),(j,I)} + x_{(i,n),(j,R)},$$

which is interpreted as the probability that a binding site is susceptible with a) age i b) degree n, and c) a partner of age j.

First, we consider the equations for the variables $x_{(i,n)(j,y)}$ and $\bar{x}_{(i,n),j}$. To derive the equations for these variables, we consider the events that can occur for susceptible binding sites, which can then be translated into the individual perspective:

(1) A susceptible binding site with an infectious partner becomes infected via sexual contact at rate β. If that happens, the binding site "leaves" the x-system (meaning that the x variables form a defective probability distribution).

(2) The infectious partner of the susceptible binding site can recover before infecting the binding site under consideration, this occurs at rate γ.

(3) A susceptible binding site can have an exposed partner; this exposed partner becomes infectious at rate σ .

(4) The susceptible individual to whom the binding site belongs can become infected through non-sexual contact, and this occurs at rate $p\lambda_i$ with

$$p\lambda_i = p \sum_{j=1}^{M} \pi_j\, c_{ij} I_j,$$

and affects each of the binding sites of the individual at the same time. Because of the independence of binding sites, the per-binding-site rate for an individual with degree n is $p\lambda_i/n = p \sum_j \pi_j\, c_{ij} I_j/n$.

(5) When a susceptible binding site is connected to a susceptible partner, the susceptible partner can get infected through either sexual contact with one of his/her other partners. The rate at which this occurs is explained below.

(6) When a susceptible binding site is connected to a susceptible partner, the susceptible partner can get infected through non-sexual contact in the population. The rate at which this occurs is explained below.

Consider event (5). Transmission through sexual contact occurs at rate β per partner. Therefore, the rate at which event (5) occurs is β multiplied by the mean number of partners $\Lambda_{(j,k)}$ of a susceptible partner v, where v has age j and degree k, and is a partner of a susceptible individual u. The

expected number of binding-site configurations by the fraction of individuals that a) are susceptible, b) are of age j, c) have degree k, and d) have a susceptible partner of age i and degree n as well as at least one infectious partner, is given by:

$$k(k-1)\chi_{(j,k),(i,n,S)}\left(\sum_{l=N_1}^{N_2}\tilde{\pi}_l\, x_{(j,k),(l,I)}\right)\left(\sum_{l=N_1}^{N_2}\tilde{\pi}_l\, \bar{x}_{(j,k),l}\right)^{k-2}.$$

In contrast, the expected number of binding-site configurations by the fraction of individuals who are susceptible, of age j and degree k, and who have a susceptible partner of age i and degree n is

$$k\chi_{(j,k),(i,n,S)}\left(\sum_{l=N_1}^{N_2}\tilde{\pi}_l\, \bar{x}_{(j,k),l}\right)^{k-1}.$$

Note that these expressions use the independence of binding sites of a susceptible individual, allowing us to translate probabilities at the binding site level to probabilities at the individual level (see [17] for details). Therefore, the expected number of infectious partners of a susceptible partner v with degree k is

$$\begin{aligned}\Lambda_{(j,k)} &= \frac{k(k-1)\chi_{(j,k),(i,n,S)}\left(\sum_{l=N_1}^{N_2}\tilde{\pi}_l\, x_{(j,k),(l,I)}\right)\left(\sum_{l=N_1}^{N_2}\tilde{\pi}_l\, \bar{x}_{(j,k),l}\right)^{k-2}}{k\chi_{(j,k),(i,n,S)}\left(\sum_{l=N_1}^{N_2}\tilde{\pi}_l\, \bar{x}_{(j,k),l}\right)^{k-1}} \\ &= (k-1)\frac{\sum_{l=N_1}^{N_2}\tilde{\pi}_l\, x_{(j,k),(l,I)}}{\sum_{m=N_1}^{N_2}\tilde{\pi}_m\, \bar{x}_{(j,k),m}}.\end{aligned}$$

Finally, we determine the rate at which event (6) occurs. Again, suppose v has degree k then the probability that v is susceptible given u is $\left(\sum_{l=N_1}^{N_2}\tilde{\pi}_l\, \bar{x}_{(j,k),l}\right)^{k-1}$. The rate at which v gets infected through non-sexual contacts is λ_j. Therefore, the rate at which (6) occurs is

$$p\lambda_j\left(\sum_{l=N_1}^{N_2}\tilde{\pi}_l\, \bar{x}_{(j,k),l}\right)^{k-1}.$$

Putting the rates for the events (1)-(6) together yields the following (infinite-dimensional) system of equations for the x system for the sexually active ages $i, j = N_1, \dots N_2$:

$$x'_{i,0} = -p\lambda_i x_{i,0},$$

and for $n \geq 1$,

$$\begin{aligned}\chi'_{(i,n),(j,k,S)} &= -\beta\Lambda_{(j,k)}\chi_{(i,n),(j,k,S)} - p\frac{\lambda_i}{n}\chi_{(i,n),(j,k,S)} - p\lambda_i\left(\sum_{l=N_1}^{N_2}\tilde{\pi}_l\, \bar{x}_{(j,k),l}\right)^{k-1} \\ x'_{(i,n),(j,E)} &= \beta\sum_{k=1}^{\infty}\Lambda_{(j,k)}\,\chi_{(i,n),(j,k,S)} + p\sum_{k=1}^{\infty}p\,\lambda_i\left(\sum_{l=N_1}^{N_2}\tilde{\pi}_l\, \bar{x}_{(j,k),l}\right)^{k-1} - p\frac{\lambda_i}{n}x_{(i,n),(j,E)} - \sigma x_{(i,n),(j,E)} \\ x'_{(i,n),(j,I)} &= \sigma x_{(i,n),(j,E)} - p\frac{\lambda_i}{n}x_{(i,n),(j,I)} - (\beta+\gamma)x_{(i,n),(j,I)} \\ \bar{x}'_{(i,n),j} &= -\beta x_{(i,n),(j,I)} - p\frac{\lambda_i}{n}\bar{x}_{(i,n),j}.\end{aligned}$$

Next, for ages $1 \leq i < N_1$ and $N_2 < i \leq M$, individuals are sexually inactive, so infection can only occur through non-sexual contacts:

$$\begin{aligned}S_i' &= -p\lambda_i S_i \\ E_i' &= p\lambda_i S_i - \sigma E_i \\ I_i' &= \sigma E_i - \gamma I_i.\end{aligned}$$

For the sexually active individuals, the equations for $S_i', E_i', I_i'$are derived as follows. An individual of age $N_1 \leq i \leq N_2$ that belongs to the sexually active group can be either of degree 0 or of degree $n > 0$. An individual of age i of degree $n > 0$ is susceptible with probability $\bar{x}_{(i,n)}^n$, where $\bar{x}_{(i,n)}^n =$

$\sum_{j=N_1}^{N_2} \tilde{\pi}_j \, \bar{x}_{(i,n),j}$. The probability that an individual is of degree n is p_n. Therefore, $S_i = p_0 x_{i,0} + \sum_{n=1}^{\infty} p_n \left(\sum_{j=N_1}^{N_2} \tilde{\pi}_j \, \bar{x}_{(i,n),j}\right)^n$. Differentiation with respect to time yields

$$\begin{aligned} S_i' &= p_0 x_{i,0}' + \sum_{n=1}^{\infty} n \, p_n \left(\sum_{j=N_1}^{N_2} \tilde{\pi}_j \, \bar{x}_{(i,n),j}\right)^{n-1} \sum_{j=N_1}^{N_2} \tilde{\pi}_j \, \bar{x}'_{(i,n),j} \\ &= -p\lambda_i S_i - \beta \sum_{n=1}^{\infty} n \, p_n \left(\sum_{j=N_1}^{N_2} \tilde{\pi}_j \, x_{(i,n),(j,I)}\right)\left(\sum_{j=N_1}^{N_2} \tilde{\pi}_j \, \bar{x}_{(i,n),j}\right)^{n-1}, \end{aligned}$$

whereas the equations for E_i', I_i' are

$$\begin{aligned} E_i' &= -S_i' - \sigma E_i \\ &= p\lambda_i S_i + \beta \sum_{n=1}^{\infty} n \, p_n \left(\sum_{j=N_1}^{N_2} \tilde{\pi}_j \, x_{(i,n),(j,I)}\right)\left(\sum_{j=N_1}^{N_2} \tilde{\pi}_j \, \bar{x}_{(i,n),j}\right)^{n-1} - \sigma E_i, \\ I_i' &= \sigma E_i - \gamma I_i. \end{aligned}$$

The `far past' conditions are $S_i(-\infty) = 1$, $x_{i,0}(-\infty) = 1$, $\bar{x}_{(i,n),j}(-\infty) = 1$, $\chi_{(i,n),(j,k,S)}(-\infty) = p_k$, and all other variables are equal to zero.

1.1 Next generation matrices

1.1.1 Epidemiological interpretation

We derive the next generation matrix (NGM) directly from the epidemiological interpretation of how new cases can be caused by one newly infected case. We prove in Section 1.1.3 below that this derivation corresponds to linearization of the system of ODE. We characterize R_0 as the dominant eigenvalue of an NGM $\boldsymbol{K}$ that contains both age and transmission route (transmission via sexual or non-sexual contact). The matrix $\boldsymbol{K}$ is therefore $2M \times 2M$ dimensional. We characterize the elements of $\boldsymbol{K}$ as follows. Let s denote transmission via sexual contact, ns transmission via non-sexual contact. An element of the matrix $\boldsymbol{K}$ is denoted by $K_{(j,y),(i,z)}$, with $i, j = 1, \dots, M$ and $y, z = s, ns$.

First of all, some elements of $\boldsymbol{K}$ are zero. A newly infected individual in ages $1 \le i < N_1, N_2 < i \le M$ can only transmit via non-sexual contact *and* his/her infector could only have transmitted via non-sexual contact. Therefore $K_{(j,ns),(i,s)} = 0 = K_{(j,s),(i,s)} = K_{(j,s),(i,ns)}$, $1 \le i < N_1, N_2 < i \le M$ and $j = 1, \dots, M$. Similarly $K_{(j,s),(i,ns)} = 0 = K_{(j,s),(i,s)}$ for $1 \le j < N_1, N_2 < j \le M$, $i = N_1 + 1, \dots, N_2$.

Now, there are the non-zero elements of $\boldsymbol{K}$. First consider a newly infected individual of age i who was infected via non-sexual contact. We are interested in the expected number $K_{(j,ns),(i,ns)}$ of secondary cases of age j caused via non-sexual contacts by a newly infected individual of age i who was himself/herself infected via non-sexual contact. The expression follows the familiar form from mass action models:

$$K_{(j,ns),(i,ns)} = \frac{p}{\gamma} c_{ij} \pi_j,$$

with $i, j = 1, \dots, M$. The expression is interpreted as follows: a newly infected individual is infectious for mean period $1/\gamma$, contacts with age group j are made at rate $c_{ij}\pi_j$, and the probability of transmission upon contact with a susceptible individual is p.

Next, we consider elements $K_{(j,ns),(i,s)}$. Note that non-sexual contacts are made independent of sexual contacts. Therefore, the expected number of secondary cases via non-sexual contact that are of age j is independent of the transmission route of the newly infected individual. Hence,

$$K_{(j,ns),(i,s)} = \frac{p}{\gamma} c_{ij} \pi_j,$$

for $i = N_1, \dots, N_2$, $j = 1, \dots, M$.

Then consider elements $K_{(j,s),(i,s)}$, $i,j = N_1, \dots, N_2$. Note that a newly infected individual who was infected via sexual contact has degree n with probability $\tilde{p}_n = np_n / \sum_{k=1}^{\infty} k\, p_k$, i.e., the size-biased degree distribution. The probability that a partner is of age j is $\tilde{\pi}_j = \pi_j / \sum_{l=N_1}^{N_2} \pi_l$. The probability that transmission to a partner occurs before the newly infected individual recovers is $\beta/(\beta+\gamma)$. Therefore,

$$K_{(j,s),(i,s)} = \frac{\beta}{\beta+\gamma} \tilde{\pi}_j \sum_{k=1}^{\infty} (k-1)\, \tilde{p}_k.$$

Finally, we consider the expected number of secondary cases $K_{(j,s),(i,ns)}$ of age j, caused via sexual contact by a newly infected individual of age i who was him/herself infected via non-sexual contact:

$$K_{(j,s),(i,ns)} = \frac{\beta}{\beta+\gamma} \tilde{\pi}_j \sum_{k=1}^{\infty} k\, p_k,$$

with $i,j = N_1, \dots, N_2$. This expression can be interpreted as follows: an individual of age i and degree k who is newly infected via non-sexual contact has k susceptible sexual partners, the probability that he/she transmits to a partner before recovery is $\frac{\beta}{(\beta+\gamma)}$, and the probability that the partner is of age j is $\tilde{\pi}_j$.

Putting all these elements together, we can decompose the NGM $\boldsymbol{K}$ into four $M \times M$ matrices:

$$\boldsymbol{K} = \begin{pmatrix} \mathbf{K}_{s,s} & \mathbf{K}_{s,ns} \\ \mathbf{K}_{ns,s} & \mathbf{K}_{ns,ns} \end{pmatrix}, \qquad (3)$$

where the four submatrices are as follows. Let the vector $\mathbf{1_S}$ be the indicator function for sexually active age groups such that $\mathbf{1_S}_i = 1$ if $i = N_1, \dots, N_2$ and 0 otherwise. Note that

$$\boldsymbol{\pi}_\mathbf{S} \mathbf{1_S}^T = \begin{pmatrix} 0 & \cdots 0 & 0 & \cdots & 0 & 0 & \cdots & 0 \\ \vdots & \ddots & \vdots & \ddots & \vdots & \vdots & \ddots & \vdots \\ 0 & \cdots 0 & 0 & \cdots & 0 & 0 & \cdots & 0 \\ 0 & \cdots 0 & \tilde{\pi}_{N_1} & \cdots & \tilde{\pi}_{N_1} & 0 & \cdots & 0 \\ \vdots & \ddots & \vdots & \ddots & \vdots & \vdots & \ddots & \vdots \\ 0 & \cdots 0 & \tilde{\pi}_{N_2} & \cdots & \tilde{\pi}_{N_2} & 0 & \cdots & 0 \\ 0 & \cdots 0 & 0 & \cdots & 0 & 0 & \cdots & 0 \\ \vdots & \ddots & \vdots & \ddots & \vdots & \vdots & \ddots & \vdots \\ 0 & \cdots 0 & 0 & \cdots & 0 & 0 & \cdots & 0 \end{pmatrix}. \qquad (4)$$

Then

$$\mathbf{K}_{s,s} = \frac{\beta}{\beta+\gamma} \sum_{k=1}^{\infty} (k-1)\, \tilde{p}_k \boldsymbol{\pi}_\mathbf{S} \mathbf{1_S}^T,$$

and

$$\mathbf{K}_{s,ns} = \frac{\beta}{\beta+\gamma} \sum_{k=1}^{\infty} k\, p_k \boldsymbol{\pi}_\mathbf{S} \mathbf{1_S}^T.$$

The submatrices involving secondary cases infected via the non-sexual route are

$$\mathbf{K}_{ns,ns} = \frac{p}{\gamma} \begin{pmatrix} c_{11}\pi_1 & c_{12}\pi_1 & \cdots & c_{1,M}\pi_1 \\ c_{21}\pi_2 & c_{22}\pi_2 & \cdots & c_{2,M}\pi_2 \\ \vdots & \vdots & \ddots & \vdots \\ c_{M,1}\pi_M & c_{M,2}\pi_M & \cdots & c_{M,M}\pi_M \end{pmatrix}, \qquad (5)$$

and

$$\mathbf{K}_{ns,s} = \frac{p}{\gamma} \begin{pmatrix} 0 & \cdots & 0 & c_{1N_1}\pi_1 & c_{1,N_1+1}\pi_1 & \cdots & c_{1N_2}\pi_1 & 0 & \cdots & 0 \\ 0 & \cdots & 0 & c_{2N_1}\pi_2 & c_{2,N_1+1}\pi_2 & \cdots & c_{2N_2}\pi_2 & 0 & \cdots & 0 \\ \vdots & \ddots & \vdots & \vdots & \vdots & \ddots & \vdots & \vdots & \ddots & \vdots \\ 0 & \cdots & 0 & c_{M2N_1}\pi_M & c_{M,N_1+1}\pi_M & \cdots & c_{M,N_2}\pi_M & 0 & \cdots & 0 \end{pmatrix}.$$

1.1.2 Reductions of the NGM

As sexual partnerships are formed independent of age, we can reduce the NGM (3) by averaging over age in NGM elements involving transmission via sexual contact. This yields the NGM

$$\widehat{\boldsymbol{K}} = \begin{pmatrix} \frac{\beta}{\beta+\gamma}\sum_{k=1}^{\infty}(k-1)\,\tilde{p}_k & \frac{\beta}{\beta+\gamma}\sum_{k=1}^{\infty}k\,p_k\mathbf{1_S} \\ \frac{p}{\gamma}\mathbf{k_{ns,s}} & \mathbf{K}_{ns,ns} \end{pmatrix}, \qquad (6)$$

where

$$\mathbf{k_{ns,s}} = \left(\pi_1 \sum_{i=N_1}^{N_2} c_{i,1}\,\tilde{\pi}_i, \dots, \pi_M \sum_{i=N_1}^{N_2} c_{i,M}\,\tilde{\pi}_i\right)^T.$$

We provide the details for this reduction of K into $\widehat{K}$ in Section 1.1.3.2.

As non-sexual contacts are age structured, i.e. the contact rates c_{ij} are dependent on i and j, we cannot further reduce the NGM. However, note that in the absence of age structure, the NGM $\widehat{\boldsymbol{K}}$ (6) can be reduced to

$$\boldsymbol{K} = \begin{pmatrix} \frac{\beta}{\beta+\gamma}\sum_{k=1}^{\infty}(k-1)\,\tilde{p}_k & \frac{\beta}{\beta+\gamma}\sum_{k=1}^{\infty}k\,p_k \\ \frac{pc}{\gamma} & \frac{pc}{\gamma} \end{pmatrix}, \qquad (7)$$

which corresponds to the NGM of (*24*) for the model without age structure. Note that the NGM (7) differs from the related model of (*23*), where a newly infected individual with n susceptible sexual partners generates an expected number of $\beta/\gamma n$ secondary cases via sexual contact rather than $\beta/(\beta+\gamma)n$ as in our model. In the absence of both age structure and non-sexual contacts, i.e. in a model with only network structure, one obtains a one-dimensional NGM equal to the basic reproduction number $\boldsymbol{K} = R_0 = \frac{\beta}{\beta+\gamma}\sum_{k=1}^{\infty}(k-1)\,\tilde{p}_k$, e.g. (*31*). Finally, in the absence of the network structure, the NGM reduces to $\mathbf{K}_{ns,ns}$ of (9), which corresponds to the NGM of an age-structured mass action SEIR model (*68*).

1.1.3 R_0 is a threshold parameter for the model

The NGM $\boldsymbol{K}$ is derived directly from the epidemiological interpretation given in Section 1.1.1. We show that the dominant eigenvalue R_0 of $\boldsymbol{K}$ is indeed a threshold parameter for the model in the sense that it determines the stability of the disease free steady state. To demonstrate this, we linearize the system around the disease free steady state, construct the NGM $\widetilde{\boldsymbol{K}}$ from the linearized system, and show that both $\widetilde{\boldsymbol{K}}$ and $\boldsymbol{K}$ can be reduced to the same NGM with small domain $\widehat{\boldsymbol{K}}$ given by (6). This proves that all three matrices $\boldsymbol{K}$, $\widetilde{\boldsymbol{K}}$ and $\widehat{\boldsymbol{K}}$ have the same dominant eigenvalue R_0. Since the dominant eigenvalue of $\widetilde{\boldsymbol{K}}$ is a threshold parameter for the disease free steady state of the model, R_0 is indeed the threshold parameter that we are after.

1.1.3.1 Linearization of the ODE system

We prove that R_0 is a threshold parameter of the system by linearization around the disease-free steady state, following the approach in (*31*). In this linearization, one finds a decoupled system for the variables E_i, I_i, $x_{(i,n),(j,E)}$, $x_{(i,n),(j,I)}$, which can be further reduced to a linearized system for the variables E_i, I_i, $\sum_{j=N_1}^{N_2} x_{(i,n),(j,E)}$, $\sum_{j=N_1}^{N_2} x_{(i,n),(j,I)}$. Additionally, in the linearization the variables $\sum_{j=N_1}^{N_2} x_{(i,n),(j,E)}$ and $\sum_{j=N_1}^{N_2} x_{(i,n),(j,I)}$ are independent of n, which allows further reduction by averaging over n. We then split the linearized system into a transmission matrix $\boldsymbol{T}$ and a transition matrix $\boldsymbol{\Sigma}$, yielding the NGM with large domain $\widetilde{\boldsymbol{K}}_L = -\boldsymbol{T}\boldsymbol{\Sigma}^{-1}$. The matrix $\widetilde{\boldsymbol{K}}_L$ can be reduced to $\widetilde{\boldsymbol{K}}$ by considering states-at-infectiousness. This yields a $2M \times 2M$ matrix. One can interpret the NGM $\widetilde{\boldsymbol{K}}$ in terms of "reproduction opportunities" (see (*31*), section 4.3).

We characterize R_0 as the dominant eigenvalue of an NGM $\widetilde{\boldsymbol{K}}$. The NGM $\widetilde{\boldsymbol{K}}$ contains both age and transmission route (transmission via sexual or non-sexual contact), and is therefore $2M \times 2M$ dimensional. We characterize the elements of $\widetilde{\boldsymbol{K}}$ as follows: let s denote transmission via sexual contact, ns transmission via non-sexual contact, elements $\widetilde{K}_{(j,y),(i,z)}$ of the matrix $\widetilde{\boldsymbol{K}}$, $i,j = 0,\dots,M$ and $y,z = s,ns$. A newly infected individual in age group $1 \le i < N_1, N_2 < i \le M$ can only transmit via non-sexual contact *and* his/her infector must also have transmitted via non-sexual contact. Therefore $\widetilde{K}_{(j,ns),(i,s)} = 0 = K_{(j,s),(i,s)} = \widetilde{K}_{(j,s),(i,ns)}$, $1 \le i < N_1, N_2 < i \le M$ and $j = 1,\dots,M$. Similarly, $\widetilde{K}_{(j,s),(i,ns)} = 0 = \widetilde{K}_{(j,s),(i,s)}$ for $1 \le j < N_1, N_2 < j \le M$, $i = N_1,\dots,N_2$. Linearization yields the following NGM elements:

$$\widetilde{K}_{(i,ns),(j,ns)} = \frac{p}{\gamma} c_{ij}\pi_j,$$

with $i,j = 1,\dots,M$,

$$\widetilde{K}_{(i,ns),(j,s)} = \frac{p}{\gamma}\pi_j c_{ji},$$

for $i = N_1,\dots,N_2$, $j = 1,\dots,M$,

$$\widetilde{K}_{(i,s),(j,s)} = \frac{\beta}{\beta+\gamma}\tilde{\pi}_j \sum_{k=1}^{\infty}(k-1)\,\tilde{p}_k,$$

for $i,j = N_1,\dots N_2$,

$$\widetilde{K}_{(j,s),(j,ns)} = \frac{\beta}{\beta+\gamma}\tilde{\pi}_j \sum_{k=1}^{\infty} k\, p_k,$$

for $j = N_1,\dots,N_2$.

Putting all these elements together, we can decompose the NGM $\boldsymbol{K}$ into four $M \times M$ submatrices:

$$\widetilde{\boldsymbol{K}} = \begin{pmatrix} \widetilde{\mathbf{K}}_{\mathbf{s,s}} & \widetilde{\mathbf{K}}_{\mathbf{s,ns}} \\ \widetilde{\mathbf{K}}_{\mathbf{ns,s}} & \widetilde{\mathbf{K}}_{\mathbf{ns,ns}} \end{pmatrix} \qquad (8)$$

where the four matrices are as follows.

$$\widetilde{\mathbf{K}}_{\mathbf{s,s}} = \frac{\beta}{\beta+\gamma}\sum_{k=1}^{\infty}(k-1)\,\tilde{p}_k \boldsymbol{\pi}_{\mathbf{S}} \mathbf{1}_{\mathbf{S}}{}^T,$$

and

$$\widetilde{\mathbf{K}}_{\mathbf{s,ns}} = \frac{\beta}{\beta+\gamma}\sum_{k=1}^{\infty} k\, p_k \boldsymbol{\pi}_{\mathbf{S}} \mathbf{1}_{\mathbf{S}}{}^T.$$

The submatrices involving secondary cases infected via non-sexual contact are

$$\widetilde{\mathbf{K}}_{\mathbf{ns,ns}} = \frac{p}{\gamma}\begin{pmatrix} c_{11}\pi_1 & c_{12}\pi_2 & \cdots & c_{1,M}\pi_M \\ c_{21}\pi_1 & c_{22}\pi_2 & \cdots & c_{2,M}\pi_M \\ \vdots & \vdots & \ddots & \vdots \\ c_{M,1}\pi_1 & c_{M,2}\pi_2 & \cdots & c_{M,M}\pi_M \end{pmatrix}, \qquad (9)$$

and

$$\widetilde{\mathbf{K}}_{\mathbf{ns,s}} = \frac{p}{\gamma}\begin{pmatrix} 0 & \cdots & 0 \\ \vdots & \ddots & \vdots \\ 0 & \cdots & 0 \\ c_{1,N_1}\pi_1 & \cdots & c_{M,N_1}\pi_M \\ \vdots & \ddots & \vdots \\ c_{1,N_2}\pi_1 & \cdots & c_{M,N_2}\pi_M \\ 0 & \cdots & 0 \\ \vdots & \ddots & \vdots \\ 0 & \cdots & 0 \end{pmatrix}.$$

1.1.3.2 Reduction to $\widehat{\boldsymbol{K}}$

We provide the calculations for the reduction of $\boldsymbol{K}$ into $\widehat{\boldsymbol{K}}$, where $\boldsymbol{K}$ and $\widehat{\boldsymbol{K}}$ are given by (3) and (6). The NGM $\widehat{\boldsymbol{K}}$ can be interpreted as the NGM with small domain of $\boldsymbol{K}$ (e.g. (*32*)). Here the reduction is in the sense that $\boldsymbol{K}$ and $\widehat{\boldsymbol{K}}$ have the same dominant eigenvalue.

Let $\boldsymbol{C}$ be the following $(1+M)\times(M+M)$ matrix:

$$\boldsymbol{C} = \begin{pmatrix} \boldsymbol{\pi} & 0 \\ 0 & I \end{pmatrix}.$$

Let $\boldsymbol{R}$ be the following $(M+M)\times(1+M)$ matrix:

$$\boldsymbol{R} = \begin{pmatrix} \frac{\beta}{\beta+\gamma}\sum_{k=1}^{\infty}(k-1)\,\tilde{p}_k \mathbf{1_S} & \mathbf{K_{ns,s}} \\ \frac{\beta}{\beta+\gamma}\sum_{k=1}^{\infty} k\, p_k \mathbf{1_S} & \mathbf{K_{ns,ns}} \end{pmatrix}.$$

Then $\boldsymbol{RC} = \boldsymbol{K}^T$ and $\boldsymbol{CR} = \widehat{\boldsymbol{K}}^T$.

Similarly, we can reduce the NGM $\widetilde{\boldsymbol{K}}$ to $\widehat{\boldsymbol{K}}$. Let $\widetilde{\boldsymbol{C}}$ be the following $(1+M)\times(M+M)$ matrix:

$$\widetilde{\boldsymbol{C}} = \begin{pmatrix} \mathbf{1_S}^T & 0 \\ 0 & \boldsymbol{I} \end{pmatrix}.$$

Let $\widetilde{\boldsymbol{R}}$ be the following $(M+M)\times(1+M)$ matrix:

$$\widetilde{\boldsymbol{R}} = \begin{pmatrix} \frac{\beta}{\beta+\gamma}\sum_{k=1}^{\infty}(k-1)\,\tilde{p}_k \boldsymbol{\pi}_\mathbf{S} & \widetilde{\mathbf{K}}_\mathbf{ns,s} \\ \frac{\beta}{\beta+\gamma}\sum_{k=1}^{\infty} k\, p_k \boldsymbol{\pi}_\mathbf{S} & \widetilde{\mathbf{K}}_\mathbf{ns,ns} \end{pmatrix}.$$

Then $\widetilde{\boldsymbol{R}}\widetilde{\boldsymbol{C}} = \widetilde{\boldsymbol{K}}$ and $\widetilde{\boldsymbol{C}}\widetilde{\boldsymbol{R}} = \widehat{\boldsymbol{K}}$.

1.1.3.3 Dominant eigenvalue

The system of ODEs describing the model of the sexual network together with non-sexual contacts can be linearized around the disease free steady state. The matrix for the linearization can be decomposed into a transmission matrix $\boldsymbol{T}$ and a transition matrix $\boldsymbol{\Sigma}$ such that $-\boldsymbol{T}\boldsymbol{\Sigma}^{-1} = \widetilde{\boldsymbol{K}}$. Since both $\widetilde{\boldsymbol{K}}$ and $\boldsymbol{K}$ have $\widehat{\boldsymbol{K}}$ as the NGM with small domain, the dominant eigenvalue R_0 of $\boldsymbol{K}$ is a threshold parameter with threshold value of one for the stability of the disease free steady state of our model. Furthermore, R_0 has the usual interpretation as the expected number of secondary cases caused by one typical newly infected individual.

2 Newly infected individuals over time

2.1 Number of sexual partners of newly infected individuals over time

Individuals of age group i become infected via non-sexual contact at rate $p\lambda_i S_i$ and via sexual contact at rate $\beta\sum_{n=1}^{\infty} n\,p_n \left(\sum_{j=N_1}^{N_2}\tilde{\pi}_j\, x_{(i,n),(j,I)}\right)\left(\sum_{j=N_1}^{N_2}\tilde{\pi}_j\, \bar{x}_{(i,n),j}\right)^{n-1}$ (see ODE for E_i).

The rate at which an individual of age i and degree n gets infected via non-sexual contact is $p_n p\lambda_i S_i$ since the route of transmission is independent of the degree. The probability that a newly infected individual of age i, infected via non-sexual contact, has degree n is

$$\begin{aligned} q_{(g,i,n)}(t) &= p_n p\lambda_i(t)S_i(t)/d_{(g,i)}(t) \\ &= p_n / \sum_k p_k \qquad (10) \\ &= p_n, \end{aligned}$$

where $d_{(g,i)}(t) := \sum_n p_n\, p\lambda_i(t)S_i(t) = p\lambda_i(t)S_i(t)$ is the (time-varying) normalizing constant.

An individual of age i and of degree n gets infected via sexual contact at rate $\beta n p_n \left(\sum_{j=N_1}^{N_2} \tilde{\pi}_j\, x_{(i,n),(j,I)}(t)\right)\left(\sum_{j=N_1}^{N_2} \tilde{\pi}_j\, \bar{x}_{(i,n),j}(t)\right)^{n-1}$. Therefore, the probability that a newly infected individual of age i who was infected via sexual contact, has degree n is given by

$$\begin{aligned} q_{(s,i,n)}(t) &= \beta n p_n \left(\sum_{j=N_1}^{N_2} \tilde{\pi}_j\, x_{(i,n),(j,I)}(t)\right)\left(\sum_{j=N_1}^{N_2} \tilde{\pi}_j\, \bar{x}_{(i,n),j}(t)\right)^{n-1} / d_{(s,i)}(t), \\ &= \frac{n p_n \left(\sum_{j=N_1}^{N_2} \tilde{\pi}_j\, x_{(i,n),(j,I)}(t)\right)\left(\sum_{j=N_1}^{N_2} \tilde{\pi}_j\, \bar{x}_{(i,n),j}(t)\right)^{n-1}}{\sum_{k=1}^{\infty} k\, p_k \left(\sum_{j=N_1}^{N_2} \tilde{\pi}_j\, x_{(i,k),(j,I)}(t)\right)\left(\sum_{j=N_1}^{N_2} \tilde{\pi}_j\, \bar{x}_{(i,k),j}(t)\right)^{k-1}}, \end{aligned} \tag{11}$$

where $d_{(s,i)}(t) := \beta \sum_{n=1}^{\infty} n\, p_n \left(\sum_{j=N_1}^{N_2} \tilde{\pi}_j\, x_{(i,n),(j,I)}(t)\right)\left(\sum_{j=N_1}^{N_2} \tilde{\pi}_j\, \bar{x}_{(i,n),j}(t)\right)^{n-1}$ is the normalizing constant.

2.2 Time-varying NGM

We derive the time-varying NGM from the interpretation similar to Section 1.1.1. With a little abuse of notation, we again use $\boldsymbol{K}$ to denote the time-varying NGM, but we now explicitly include the time dependence: $\boldsymbol{K}(t)$.

First, consider a newly infected individual of age i who was infected via non-sexual contact. We are interested in the expected number $K_{(j,ns),(i,ns)}(t)$. We now need to take into account the fraction of susceptible individuals of age j at time t:

$$K_{(j,ns),(i,ns)}(t) = \frac{p}{\gamma} c_{ij} \pi_j S_j(t),$$

with $i, j = 1, \dots, M$. Similarly,

$$K_{(j,ns),(i,s)}(t) = \frac{p}{\gamma} c_{ij} \pi_j S_j(t),$$

for $i = N_1, \dots, N_2$, $j = 1, \dots, M$.

Next, we consider a newly infected individual of age i, infected via sexual contact, and derive the expected number of secondary cases $K_{(j,s),(i,s)}(t)$, $i, j = N_1, \dots, N_2$. At time t, a newly infected individual who was infected via sexual contact has degree n with probability $q_{(s,i,n)}(t)$ (see (11)). At the moment of infection, partners are independent of one another. We can consider the probability that a partner of age j of a newly infected individual has degree k. This probability is given by the size-biased degree distribution $\tilde{p}_k$. He/she is susceptible at time t with probability $x_{(j,k),(i,S)}(t)\left(\sum_{l=N_1}^{N_2} \tilde{\pi}_l\, \bar{x}_{(j,k)}(t)\right)^{k-1}$ (one of his/her k partners was recently susceptible and has age i, the other $k-1$ binding sites are susceptible). Therefore, the probability that a partner of age j of a newly infected individual of age i at time t is susceptible is equal to

$$P_{S_{i,j}}(t) := \sum_{k=0}^{\infty} \tilde{p}_k\, x_{(j,k),(i,S)}(t) \left(\sum_{l=N_1}^{N_2} \tilde{\pi}_l\, \bar{x}_{(j,k)}(t)\right)^{k-1}.$$

Note that $P_{S_{ij}}(-\infty) = 1$, corresponding to all partners being susceptible in the disease-free steady state. The time-dependent NGM element $K_{(j,s),(i,s)}(t)$ is:

$$K_{(j,s),(i,s)}(t) = \frac{\beta}{\beta + \gamma} \tilde{\pi}_j P_{S_{i,j}}(t) \sum_{k=1}^{\infty} (k-1)\, q_{(s,i,k)}(t).$$

Finally, the expected number of secondary cases $K_{(j,s),(i,ns)}(t)$ is:

$$\begin{aligned} K_{(j,s),(i,g)}(t) &= \frac{\beta}{\beta+\gamma}\tilde{\pi}_j P_{S_{i,j}}(t) \sum_{k=1}^{\infty} k\, q_{(ns,i,k)}(t) \\ &= \frac{\beta}{\beta+\gamma}\tilde{\pi}_j P_{S_{i,j}}(t) \sum_{k=1}^{\infty} k\, p_k, \end{aligned}$$

with $i, j = N_1, \dots, N_2$, and $q_{(ns,i,k)}$ is given by (10).

As in Section 1.1.1, all other elements are equal to zero. Putting everything together in a matrix of the form (3) we obtain the time-varying NGM $\boldsymbol{K}(t)$.

2.3 Mean degree of a newly infected individual over time

To obtain the mean number of partners of a newly infected individual, we proceed as follows. Averaging over age yields the rate at which an individual of degree n gets infected:

$$q_n(t) = \sum_{i=N_1}^{N_2} \tilde{\pi}_i \left(p_n p \lambda_i(t) S_i(t) + \beta n p_n \left(\sum_{j=N_1}^{N_2} \tilde{\pi}_j\, x_{(i,n),(j,I)}(t) \right) \left(\sum_{j=N_1}^{N_2} \tilde{\pi}_j\, \bar{x}_{(i,n),j}(t) \right)^{n-1} \right).$$

By normalizing this distribution $\left(q_n(t)\right)_n$ and averaging over n, we obtain the mean degree of a newly infected individual at time t.

3 Perturbation analysis of NGM

3.1 Framework

In the following sections, we quantify the expected impact of targeted interventions on the time-varying NGM $\mathbf{K}(t)$ (Section 2.2) and consequently on its dominant eigenvalue $R(t)$. From hereon, we drop explicit writing of t and use $\mathbf{K}$ and R for notational simplicity. The general principle is to identify the group that yields the largest expected reduction in R when a single unit of vaccines is allocated to that group. We begin by introducing the sensitivity and elasticity of matrices and their epidemiological interpretations. We then formulate the expected change in the NGM $\mathbf{K}$ for different targeted vaccination strategies. This approach has been widely used in demography and ecology (*53*), and as a tool to analyze the NGM in epidemiology (*32*, *33*, *34*, *52*). Here, we focus on vaccine allocation to illustrate this framework, although it can be extended to other interventions such as school or workplace closures.

3.2 Sensitivity and elasticity of NGM

Targeted interventions influence the entries of the NGM $\mathbf{K}$. We denote the matrix entries of $\mathbf{K}$ by k_{mn}, with $m, n = 1, \dots, 2M$, representing all combinations of age (across M age groups) and transmission route (sexual s or non-sexual ns). The sensitivity of R with respect to a change in entry k_{mn} is expressed as

$$[\mathcal{S}]_{mn} = \frac{\Delta R}{\Delta k_{mn}},$$

and the elasticity is given by

$$[\mathcal{E}]_{mn} = \frac{k_{mn}}{R} \frac{\Delta R}{\Delta k_{mn}},$$

or in matrix form

$$\mathcal{E} = \frac{1}{R} \mathbf{K} \odot \mathcal{S},$$

where $\odot$ represents the Hadamard (element-wise) product. The cumulative elasticity for the column ℓ is obtained by summing over the elasticities $[\mathcal{E}]_{m\ell}$ across all groups m:

$$e_\ell = \sum_{m=1}^{2M} \frac{k_{m\ell}}{R} \frac{\Delta R}{\Delta k_{m\ell}} = \sum_{m=1}^{2M} \frac{k_{m\ell}}{R} [\mathcal{S}]_{m\ell} = \sum_{m=1}^{2M} [\mathcal{E}]_{m\ell}.$$

The cumulative elasticity e_ℓ measures the proportional contribution of column ℓ to the change in R given the same perturbation to every entry k_{mn}. If we apply it to an age-stratified NGM with a single transmission route, the cumulative elasticity can be interpreted as the total contribution from a specific age group to the overall transmission potential R.

In our setting, an NGM element represents the age- and transmission route-specific number of secondary infections. We define the age-specific contribution to R by summing the cumulative elasticities of age group i for two transmission routes:

$$\tilde{e}_i = e_{(\mathrm{i,s})} + e_{(\mathrm{i,ns})}.$$

Similarly, we define the cumulative sensitivity of age group i as $\tilde{s}_i$.

3.3 Targeted vaccination

3.3.1 Number of vaccines, vaccination coverage, and effective vaccination coverage

Let $u_{i,n}$ denote the number of vaccines (i.e., the number of individuals who complete the full vaccine series) allocated to the group of age i with degree n (hereafter called the group (i, n)). The total number of vaccines allocated to the age group i is given by:

$$u_i = \sum_n u_{i,n},$$

and the total number of vaccines allocated across the entire population is

$$U = \sum_i u_i = \sum_i \left(\sum_n u_{i,n} \right).$$

We define the vaccination coverage for group (i, n) as the proportion of vaccinated individuals:

$$v_{i,n} = \frac{u_{i,n}}{S_{i,n} p_n \pi_i N},$$

where $S_{i,n}$ is the proportion susceptible in group (i, n) and N is the total population. By considering vaccine efficacy $\varepsilon_{i,n}$ (VE) against infection in group (i, n), $\varepsilon_{i,n}$the proportion of effectively vaccinated individuals (i.e., effective vaccination coverage) in group (i, n) is given by $\varepsilon_{i,n} v_{i,n}$. In the present study, we assume a uniform VE for all age groups: $\varepsilon_{i,n} = \varepsilon$.

3.3.2 Impact of targeted vaccination strategies

Here, we consider the unit change in the total number ΔU of vaccines allocated. We compute the unit change $\Delta v_{i,n}$ in the vaccination coverage in group (i, n), if the single unit of vaccines is allocated to that group.

If the vaccination is at random, vaccines will be allocated proportional to the relative population size of the age group i:

$$\Delta u_{i,n} = p_n \pi_i \Delta U.$$

The unit change in the vaccination coverage is given by

$$\Delta v_{i,n} = \frac{\Delta u_{i,n}}{S_{i,n} p_n \pi_i N} = \frac{\Delta U}{S_{i,n} N},$$

where p_n is the proportion with degree n, π_i is the proportion of age i, $S_{i,n}$ is the proportion of suscepibles in age group i with degree n, and N is the total population size. This represents the situation where all groups (i, n) receive the same number of vaccines. The unit change in the vaccination coverage $\Delta v_{i,n}$ is further used to derive an NGM under vaccination (see section 3.4 below).

3.4 NGM with vaccination

3.4.1 Change in the proportion of susceptibles in group (i, n)

Using the unit change in the effective vaccination coverage $\varepsilon \Delta v_{i,n}$, the unit change in the proportion of susceptibles in group (i, n) is expressed as:

$$\Delta\bar{x}_{(i,n),j} = \varepsilon\Delta v_{i,n}\bar{x}_{(i,n),j}$$

and this holds for any partner of age group j. In the next section, we relate this unit change to the change in elements of the NGM and quantify the cumulative sensitivity and elasticity.

3.4.2 Change in the elements of the NGM

Let $q^v_{(s,i,k)}(t)$ denote the probability that a newly infected individual of age i infected through sexual contact (s) has degree n under vaccination (v). First, if age group i is targeted for vaccination, the probability that a binding site is susceptible with age i and degree n and has a partner of age j after a unit increase in effective vaccination coverage is given by:

$$\begin{aligned}\bar{x}^v_{(i,n),j} &:= \bar{x}_{(i,n),j} - \Delta\bar{x}_{(i,n),j}\\ &= \left(1-\varepsilon\Delta v_{i,n}\right)x_{(i,n),(j,S)} + \left(1-\varepsilon\Delta v_{i,n}\right)x_{(i,n),(j,E)} + \left(1-\varepsilon\Delta v_{i,n}\right)x_{(i,n),(j,I)}\\ &\quad + \left(1-\varepsilon\Delta v_{i,n}\right)x_{(i,n),(j,R)}\end{aligned}$$

because the vaccine reduces the proportion of susceptibles in age i, independent from the partner's infection status (i.e., the same reasoning for computing $P_{S_{i,j}}$).

Second, this change leads to the change of $q^v_{(s,i,k)}(t)$, and it is expressed as:

$$q^v_{(s,i,n)}(t) = \frac{\beta n p_n \left(\sum_{j=N_1}^{N_2} \tilde{\pi}_j\, x^v_{(i,n),(j,I)}(t)\right)\left(\sum_{j=N_1}^{N_2} \tilde{\pi}_j\, \bar{x}^v_{(i,n),j}(t)\right)^{n-1}}{d^v_{(s,i)}(t)}.$$

By substituting $\left(1-\varepsilon\Delta v_{i,n}\right)x_{(i,n),(j,I)}$ and $\left(1-\varepsilon\Delta v_{i,n}\right)\bar{x}_{(i,n),j}$ to $x^v_{(i,n),(j,I)}(t)$ and $\bar{x}^v_{(i,n),j}(t)$, $q^v_{(s,i,k)}(t)$ is rearranged as

$$q^v_{(s,i,n)}(t) = \frac{\beta n p_n \left(1-\varepsilon\Delta v_{i,n}\right)^n \left(\sum_{j=N_1}^{N_2} \tilde{\pi}_j\, x_{(i,n),(j,I)}(t)\right)\left(\sum_{j=N_1}^{N_2} \tilde{\pi}_j\, \bar{x}_{(i,n),j}(t)\right)^{n-1}}{d^v_{(s,i)}(t)},$$

and $d^v_{(s,i)}(t)$ is rearranged as

$$d^v_{(s,i)}(t) = \beta \sum_{n=1}^{\infty} n\, p_n \left(1-\varepsilon\Delta v_{i,n}\right)^n \left(\sum_{j=N_1}^{N_2} \tilde{\pi}_j\, x_{(i,n),(j,I)}(t)\right)\left(\sum_{j=N_1}^{N_2} \tilde{\pi}_j\, \bar{x}_{(i,n),j}(t)\right)^{n-1}.$$

For age groups that are not targeted, the probabilities $q_{(s,i,n)}(t)$ remain the same.

Next, we consider $P^v_{S_{i,j}}(t)$, the probability that a partner of age j of an individual in age group i is susceptible after vaccination. If age group j is not targeted, the probability remains unchanged, i.e., $P^v_{S_{i,j}}(t) = P_{S_{i,j}}(t)$. If age group j is targeted, then:

$$P^v_{S_{i,j}}(t) := \sum_{k=0}^{\infty} \tilde{p}_k\, x^v_{(j,k),(i,E)}(t) \left(\sum_{l=N_1}^{N_2} \tilde{\pi}_l\, \bar{x}^v_{(j,k)}(t)\right)^{k-1}.$$

By using $x^v_{(j,k),(i,E)}(t) = \left(1-\varepsilon\Delta v_{j,k}\right)x_{(j,k),(i,E)}(t)$ and $\bar{x}^v_{(j,k)}(t) = \left(1-\varepsilon\Delta v_{j,k}\right)\bar{x}_{(j,k)}(t)$, we obtain

$$P^v_{S_{i,j}}(t) = \sum_{k=0}^{\infty} \tilde{p}_k\, \left(1-\varepsilon\Delta v_{i,n}\right)x_{(j,k),(i,E)}(t) \left(\sum_{l=N_1}^{N_2} \tilde{\pi}_l\, \left(1-\varepsilon\Delta v_{i,n}\right)\bar{x}_{(j,k)}(t)\right)^{k-1}.$$

Lastly, for transmission via non-sexual contact, only the proportion of susceptibles in age group i is affected. The updated proportion of susceptibles after vaccination is:

$$S^v_i := \left(1 - \varepsilon \sum_n \Delta v_{i,n}\right) S_i$$

Using the result of section 2.2, the changes in susceptible-related terms (i.e., $q^{v}_{(s,i,k)}(t)$, $P^{v}_{S_{i,j}}(t)$, and S^{v}_{i}) directly propagate into the updated NGM after vaccination, $\mathbf{K}^{v}$, which incorporates the effect of vaccination on transmission potential. From this, one can compute the post-vaccination sensitivities and elasticities of the system, analogous to the framework described in Section 3.2.

Supplementary Figures

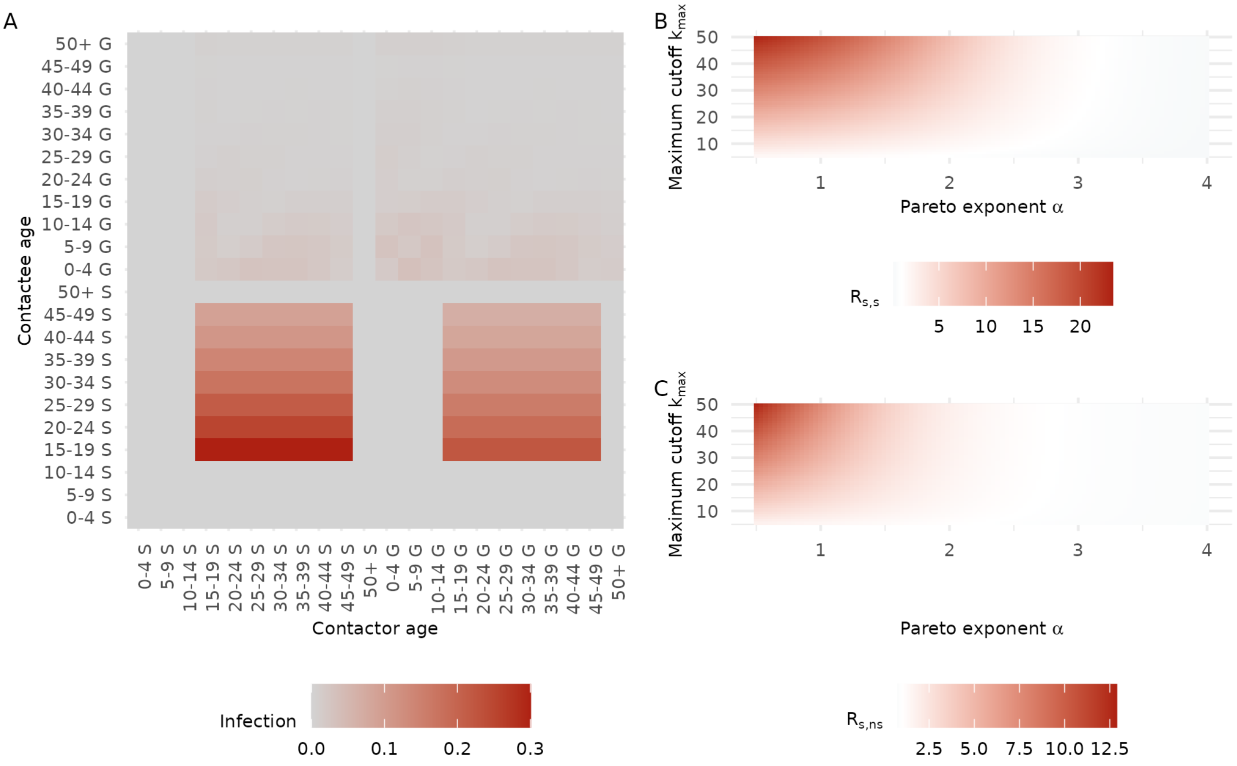

Figure S1. ***Projected next-generation matrix and reproduction numbers by transmission route and degree distribution parameters.*** (A) Full next-generation matrix (NGM) showing transmission via sexual and non-sexual (denoted with s and ns) contacts, stratified by age group. (B) Projected range of from-sexual-to-sexual reproduction numbers ($R_{s,s}$) and (C) from-non-sexual-to-sexual reproduction numbers ($R_{s,ns}$) as functions of the Pareto exponent (lower values indicate heavier-tailed degree distributions) and the maximum cutoff (i.e., the maximum number of sexual partners).

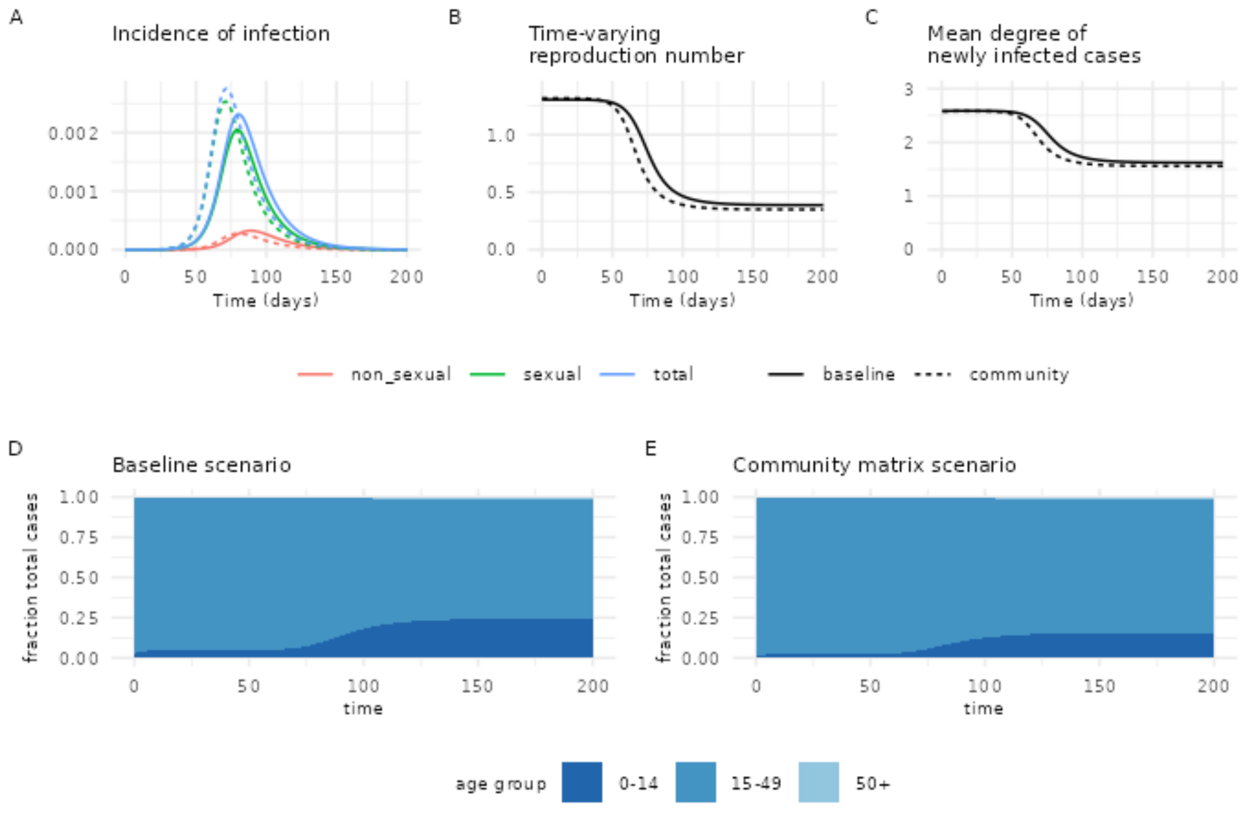

Figure S2. ***Comparison of baseline and community contact matrix scenarios.*** The baseline scenario uses a household contact matrix, and the community contact matrix scenario accounts for all contacts both within and outside households. (A) Incidence of infection per day by route of transmission. (B) Time-varying reproduction number. (C) Mean number of partners of a newly infected case (D, E) Age-distribution of infection over time for the baseline scenario (D) and the community matrix scenario (E).

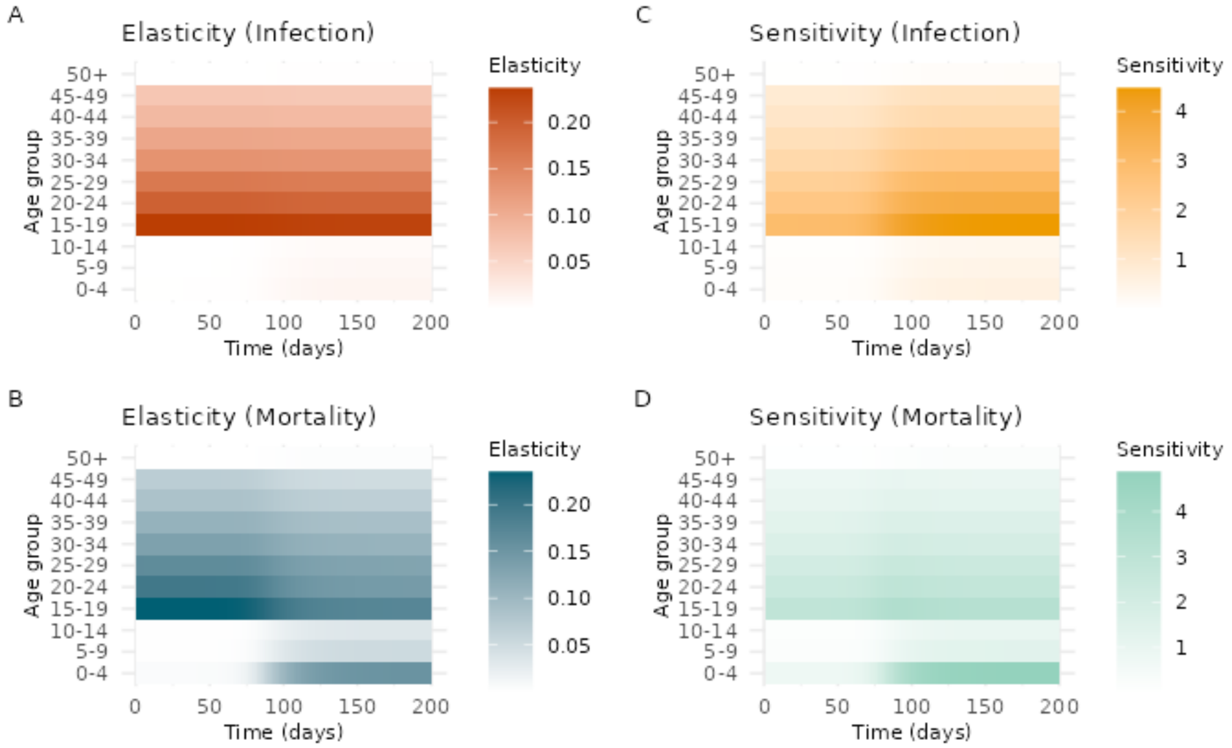

Figure S3. ***Projected age-specific sensitivity and elasticity for infection and mortality over time.*** Elasticity and sensitivity are calculated from the next-generation matrix (NGM) for infection and a mortality-weighted NGM for death. (A) Age-specific elasticity for infection. (B) Age-specific elasticity for mortality. (C) Age-specific sensitivity for infection. (D) Age-specific sensitivity for mortality. Elasticity and sensitivity values for each age group are to be compared relative to each other – higher elasticity or sensitivity means higher contribution of that age group. Compare to Figure 2 C-F in the main text, where elasticity (Fig. 2 C,E vs Fig. S3 A,B) and sensitivity (Fig. 2 D,F vs Fig. S3 C,D) for infection and mortality at time t=0 and t=200 are displayed.

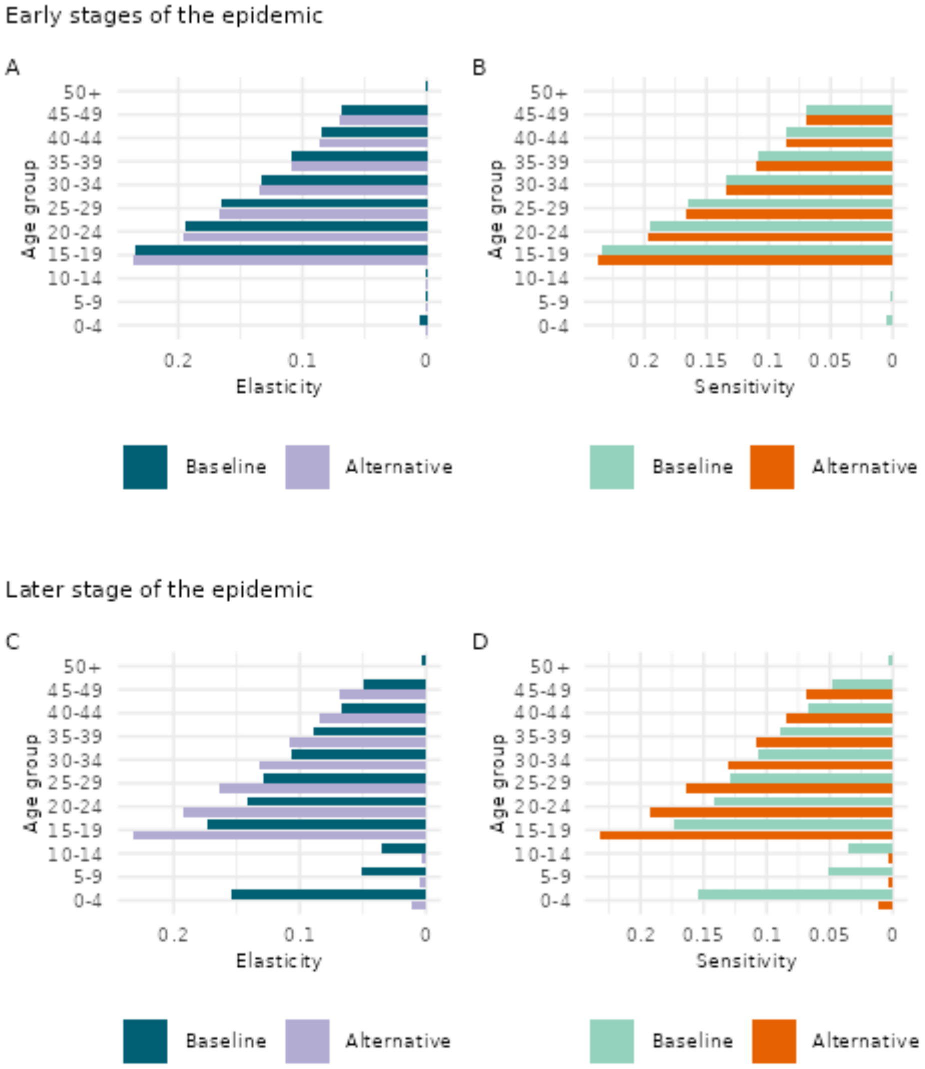

Figure S4. ***Comparison of the projected age-specific elasticity and sensitivity for mortality for the baseline mortality matrix and alternative mortality matrix.*** Elasticity and sensitivity are calculated from the mortality-weighted NGM for death. Alternative mortality matrix is based on the case fatality ratio in South Kivu reported by the Democratic Republic of Congo Ministry of Public Health (0.2% for 0-4 yo, 0.1% for 5-14 yo, and 0.2% for 15+) (*3*). (A, B) Age-specific elasticity and sensitivity for mortality at the early stages of the epidemic (t = 0). (C, D) Age-specific elasticity and sensitivity for mortality at later stage of the epidemic (t = 200). Elasticity and sensitivity for the baseline mortality are also displayed in Figure 2 C-F in the main text.

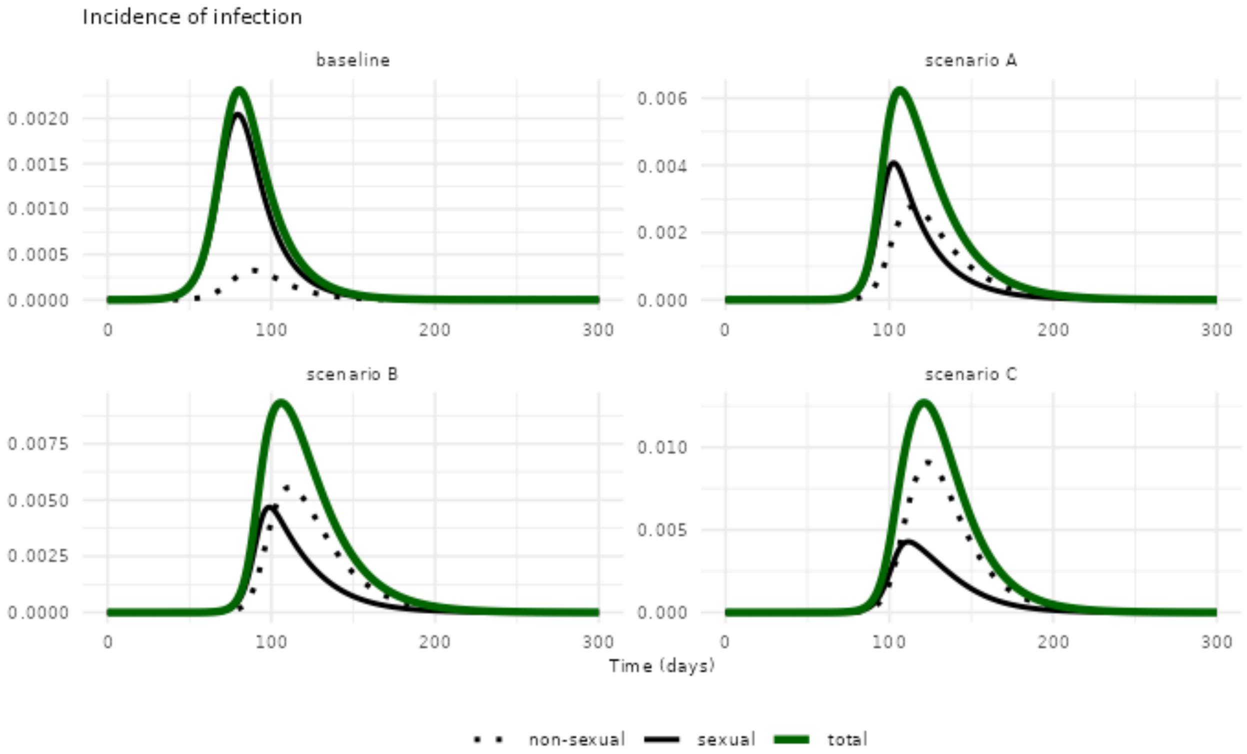

Figure S5. ***Projected incidence of infection by route of transmission.*** Comparing the baseline scenario with scenarios A, B and C where secondary attack risks of household and sexual contact are varied (refer to Table 1 in the main text).

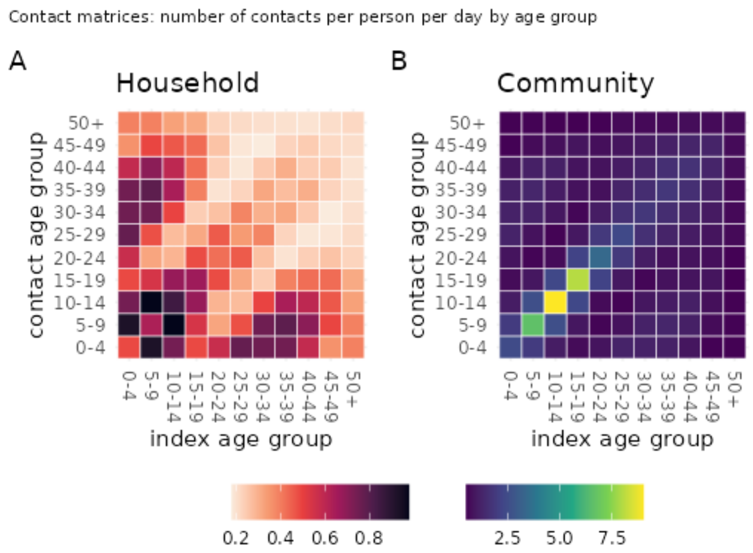

Figure S6. *Synthetic contact matrices for the Democratic Republic of Congo*. The number of contacts per person per day by age group in household setting (A) and in community (B). Household contact matrix is used for the baseline scenario, and the community contact matrix is used in the sensitivity analysis in Figure S2.

Table S1. ***Baseline values and explored ranges of model parameters.***

Parameter	Symbol	Baseline	Range	Unit	Reference
Sexually inactive proportion	N_0	0.14	0.12–0.2	-	(*45*)
Power-law exponent	α	2.9	2.9–3.9	-	(*56*)
Maximum cutoff	$k_{\max}$	25	25	-	(*57*)
Latent period	$1/\sigma$	2	0–2	days	(*59*)
Infectious period	$1/\gamma$	8	7–17	days	(*60*)
Household SAR*	ϕ_H	0.10	0–0.2	-	(*41*)
Sexual SAR*	ϕ_S	0.8	0.3–0.95	-	(*59*)
Age-specific infection mortality ratio†: 0-4 year olds	M_{11}	0.175	-	-	(*10*)
Age-specific infection mortality ratio †: 5-14 year olds	M_{22}, M_{33}	0.05	-	-	(*10*)
Age-specific infection mortality ratio †: 15+ year olds	$M_{ii}, i \geq 4$	0.025	-	-	(*10*)

*SAR: Secondary attack risk

†Age-specific infection mortality ratio is determined using the reported case-fatality ratio in as a proxy.

REFERENCES

1. E. H. Vakaniaki, C. Kacita, E. Kinganda-Lusamaki, Á. O'Toole, T. Wawina-Bokalanga, D. Mukadi-Bamuleka, A. Amuri-Aziza, N. Malyamungu-Bubala, F. Mweshi-Kumbana, L. Mutimbwa-Mambo, F. Belesi-Siangoli, Y. Mujula, E. Parker, P.-C. Muswamba-Kayembe, S. S. Nundu, R. S. Lushima, J.-C. Makangara-Cigolo, N. Mulopo-Mukanya, E. Pukuta-Simbu, P. Akil-Bandali, H. Kavunga, O. Abdramane, I. Brosius, E. Bangwen, K. Vercauteren, N. A. Sam-Agudu, E. J. Mills, O. Tshiani-Mbaya, N. A. Hoff, A. W. Rimoin, L. E. Hensley, J. Kindrachuk, C. Baxter, T. de Oliveira, A. Ayouba, M. Peeters, E. Delaporte, S. Ahuka-Mundeke, E. L. Mohr, N. J. Sullivan, J.-J. Muyembe-Tamfum, J. B. Nachega, A. Rambaut, L. Liesenborghs, P. Mbala-Kingebeni, Sustained human outbreak of a new MPXV clade I lineage in eastern Democratic Republic of the Congo. *Nat. Med.* **30**, 2791–2795 (2024).

2. N. Ndembi, M. O. Folayan, A. Komakech, K. Mercy, S. Tessema, P. Mbala-Kingebeni, C. Ngandu, N. Ngongo, J. Kaseya, S. S. Abdool Karim, Evolving epidemiology of mpox in Africa in 2024. *N. Engl. J. Med.* **392**, 666–676 (2025).

3. World Health Organization, 2022-25 Mpox Outbreak: Global Trends (2025), https://worldhealthorg.shinyapps.io/mpx_global/.

4. WHO Rapid Risk Assessment - Mpox, Global v.3 (2025), https://who.int/publications/m/item/who-rapid-risk-assessment---mpox--global-v.3.

5. European Centre for Disease Prevention and Control, Transmission of monkeypox virus clade I: Overall risk remains low in the EU/EEA (2025), https://ecdc.europa.eu/en/news-events/transmission-monkeypox-virus-clade-i-overall-risk-remains-low-eueea.

6. CDC, Mpox in the United States and Around the World: Current Situation (2025), https://cdc.gov/mpox/situation-summary/index.html.

7. E. M. Bunge, B. Hoet, L. Chen, F. Lienert, H. Weidenthaler, L. R. Baer, R. Steffen, The changing epidemiology of human monkeypox—A potential threat? A systematic review. *PLoS Negl. Trop. Dis.* **16**, e0010141 (2022).

8. C. Kremer, S. S. Nundu, E. H. Vakaniaki, I. Brosius, G. Mukari, P. Munganga, E. Bangwen, J. C. Tshomba, Y. Mujula, E. De Vos, C. Van Dijck, S. Houben, O. Gressani, C. Kacita, D. Mukadi-Bamuleka, T. Wawina, E. Lusamaki, A. Aziza, P. Mutombo, J.-J. Muyembe-Tamfum, P. Mbala-Kingebeni, L. Liesenborghs, N. Hens, A. Torneri, Epidemiological characteristics of mpox virus Clade Ib in the Democratic Republic of the Congo: The impact of transmission mode. medRxiv 25328406 [Preprint] (2025). https://doi.org/10.1101/2025.05.27.25328406.

9. J. Perez-Saez, P. M. Bugeme, M. O'Driscoll, P. K. Bugale, Trust Faraja Mukika, L. Bugwaja, S. M. Shangula, J. Bengehya, S. Ngai, A. I. Carrion Martin, J. Jackson, N. Mulopo-Mukanya, J. Knee, I. Eckerle, E. C. Lee, D. Mukadi-Bamuleka, J. Lessler, A. S. Azman, E. B. Malembaka, The incubation periods of Monkeypox virus clade Ib. medRxiv 25322865 [Preprint] (2025) https://doi.org/10.1101/2025.02.25.25322865.

10. L. K. Whittles, P. Mbala-Kingebeni, N. M. Ferguson, Age-patterns of severity of clade I mpox in historically endemic countries. medRxiv 24306209 [Preprint] (2024). https://doi.org/10.1101/2024.04.23.24306209.

11. L. M. Masirika, J. C. Udahemuka, L. Schuele, P. Ndishimye, S. Otani, J. B. Mbiribindi, J. M. Marekani, L. M. Mambo, N. M. Bubala, M. Boter, D. F. Nieuwenhuijse, T. Lang, E. B. Kalalizi, J. P. Musabyimana, F. M. Aarestrup, M. Koopmans, B. B. Oude Munnink, F. B. Siangoli, Ongoing mpox outbreak in Kamituga, South Kivu province, associated with monkeypox virus of a novel Clade I sub-lineage, Democratic Republic of the Congo, 2024. *Euro Surveill.* **29**, 2400106 (2024).

12. I. Brosius, E. H. Vakaniaki, G. Mukari, P. Munganga, J. C. Tshomba, E. De Vos, E. Bangwen, Y. Mujula, A. Tsoumanis, C. Van Dijck, A. Alengo, L. Mutimbwa-Mambo, F. M. Kumbana, J. B. Munga, D. M. Mambo, J. W. Zangilwa, S. B. Kitwanda, S. Houben, N. A. Hoff, J.-C. Makangara-Cigolo, E. Kinganda-Lusamaki, M. Peeters, A. W. Rimoin, J. Kindrachuk, N. Low, P. D. M. C. Katoto, E. B. Malembaka, J. H. Amuasi, O. Tshiani-Mbaya, D. M. Kambaji, R. Kojan, C. Kacita, D. Mukadi-Bamuleka, S. Ahuka-Mundeke, K. Vercauteren, T. Wawina-Bokalanga, J.-J. Muyembe-Tamfum, S. S. Nundu, L. Liesenborghs, P. Mbala-Kingebeni, Epidemiological and clinical features of mpox during the clade Ib outbreak in South Kivu, Democratic Republic of the Congo: A prospective cohort study. *Lancet* **405**, 547–559 (2025).

13. J. O. Otshudiema, L. Nkengurutse, G. Kamwenubusa, I. Diallo, A. Sibomana, O. Nsavyimana, S. Harakandi, J. C. Mbonicura, J. C. Nkurunziza, F. Cishahayo, D. Niyongere, B. Havyarimana, D. Simbarariye, M. Nimburanira, B. Ntiranyibagira, A. Bitaneza, Q. Irankunda, A. B. Kanyange, B. Nikoyandemye, S. Nduwimana, F. Nyabenda, O. Kamatari, S. Irambona, A. Niyomwungere, J. T. Ntwari, A. Bousso, S. Boland, P. R. Otim, R. F. Lewis, A. Zumla, J. Nyandwi, Epidemiological characteristics and transmission dynamics of mpox in Bujumbura, Burundi: A prospective cohort study. *Soc. Sci. Res. Netw.*, https://doi.org/10.2139/ssrn.5144118 (2025).

14. A. M. Vaughan, O. Cenciarelli, S. Colombe, L. Alves de Sousa, N. Fischer, C. M. Gossner, J. Pires, G. Scardina, G. Aspelund, M. Avercenko, S. Bengtsson, P. Blomquist, A. Caraglia, E. Chazelle, O. Cohen, A. Diaz, C. Dillon, I. Dontsenko, K. Kotkavaara, M. Fafangel, F. Ferraro, R. Firth, J. Fonager, C. Frank, M. G. Carrasco, K. Gkolfinopoulou, M. P. Grenersen, B. R. Guzmán Herrador, J. Henczkó, E. Hoornenborg, D. Igoe, M. Ilić, K. Jansen, D.-G. Janță, T. B. Johansen, A. Kasradze, A. Koch, J. Kyncl, J. V. Martins, A. McAuley, K. Mellou, Z. Molnár, Z. Mor, J. Mossong, A. Novacek, H. Orlikova, I. P. Novosel, M. K. Rossi, M. Sadkowska-Todys, C. Sawyer, D. Schmid, A. Sîrbu, K. Sondén, A. Tarantola, M. Tavares, M. Thordardottir, V. Učakar, C. Van Ewijk, J. Varjas, A. Vergison, R. Vivancos, K. Zakrzewska, R. Pebody, J. M. Haussig, A large multi-country outbreak of monkeypox across 41 countries in the WHO European Region, 7 March to 23 August 2022. *Euro Surveill.* **27**, 2200620 (2022).

15. K. A. Wendorf, R. Ng, C. Stainken, M. Haddix, E. Peterson, J. Watson, D. Sachdev, Household transmission of mpox to children and adolescents, California, 2022. *J. Infect. Dis.* **229**, S203–S206 (2024).

16. J. B. Kangbai, E. Saidu, I. K. Foday, E. S. Kamanda, M. Jaba, C. M. Ruis, L. Subissi, M. Halbrook, S. Merritt, J.-C. Makangara-Cigolo, E. Kinganda Lusamaki, L. Liesenborghs, I. Bogoch, S. Y. Shaw, M. Placide, A. W. Rimoin, J. Kindrachuk, Clinical and epidemiological characteristics among probable and confirmed patients with mpox in Sierra Leone reported from January to May 2025. medRxiv 25328691 [Preprint] (2025). https://doi.org/10.1101/2025.05.30.25328691.

17. S. E. Mate, J. R. Kugelman, T. G. Nyenswah, J. T. Ladner, M. R. Wiley, T. Cordier-Lassalle, A. Christie, G. P. Schroth, S. M. Gross, G. J. Davies-Wayne, S. A. Shinde, R. Murugan, S. B. Sieh, M. Badio, L. Fakoli, F. Taweh, E. de Wit, N. van Doremalen, V. J. Munster, J. Pettitt, K. Prieto, B. W. Humrighouse, U. Ströher, J. W. DiClaro, L. E. Hensley, R. J. Schoepp, D. Safronetz, J. Fair, J. H. Kuhn, D. J. Blackley, A. S. Laney, D. E. Williams, T. Lo, A. Gasasira, S. T. Nichol, P. Formenty, F. N. Kateh, K. M. De Cock, F. Bolay, M. Sanchez-Lockhart, G. Palacios, Molecular evidence of sexual transmission of Ebola virus. *N. Engl. J. Med.* **373**, 2448–2454 (2015).

18. E. D'Ortenzio, S. Matheron, Y. Yazdanpanah, X. de Lamballerie, B. Hubert, G. Piorkowski, M. Maquart, D. Descamps, F. Damond, I. Leparc-Goffart, Evidence of sexual transmission of Zika virus. *N. Engl. J. Med.* **374**, 2195–2198 (2016).

19. J. L. Abbate, C. L. Murall, H. Richner, C. L. Althaus, Potential impact of sexual transmission on Ebola virus epidemiology: Sierra Leone as a case study. *PLOS Negl. Trop. Dis.* **10**, e0004676 (2016).

20. H. Lee, H. Nishiura, Sexual transmission and the probability of an end of the Ebola virus disease epidemic. *J. Theor. Biol.* **471**, 1–12 (2019).

21. W. Valega-Mackenzie, K. R. Ríos-Soto, Can vaccination save a Zika virus epidemic? *Bull. Math. Biol.* **80**, 598–625 (2018).

22. H. Murayama, T. R. Asakura, B. L. Dickens, J. H. Foo, S. Jin, P. K. Mukadi, K. Ejima, S.-M. Jung, A. Nishi, K. Prem, A. Endo, Roles of community and sexual contacts as drivers of clade I mpox outbreaks. medRxiv 24315554 [Preprint] (2025). https://doi.org/10.1101/2024.10.15.24315554.

23. P. K. Kollepara, R. H. Chisholm, J. C. Miller, Heterogeneity in network structure switches the dominant transmission mode of infectious diseases. *PNAS Nexus* **2**, pgad227 (2023).

24. F. Ball, P. Neal, Network epidemic models with two levels of mixing. *Math. Biosci.* **212**, 69–87 (2008).

25. M. Bellerose, L. Zhu, L. M. Hagan, W. W. Thompson, L. M. Randall, Y. Malyuta, J. A. Salomon, B. P. Linas, A review of network simulation models of hepatitis C virus and HIV among people who inject drugs. *Int. J. Drug Policy* **88**, 102580 (2021).

26. J. C. Miller, Mathematical models of SIR disease spread with combined non-sexual and sexual transmission routes. *Infect. Dis. Model.* **2**, 35–55 (2017).

27. A. Allard, B. M. Althouse, S. V. Scarpino, L. Hébert-Dufresne, Asymmetric percolation drives a double transition in sexual contact networks. *Proc. Natl. Acad. Sci. U.S.A.* **114**, 8969–8973 (2017).

28. A. Allard, B. M. Althouse, L. Hébert-Dufresne, S. V. Scarpino, The risk of sustained sexual transmission of Zika is underestimated. *PLOS Pathog.* **13**, e1006633 (2017).

29. M. Keeling, The implications of network structure for epidemic dynamics. *Theor. Popul. Biol.* **67**, 1–8 (2005).

30. L. Pellis, F. Ball, S. Bansal, K. Eames, T. House, V. Isham, P. Trapman, Eight challenges for network epidemic models. *Epidemics* **10**, 58–62 (2015).

31. K. Y. Leung, O. Diekmann, Dangerous connections: On binding site models of infectious disease dynamics. *J. Math. Biol.* **74**, 619–671 (2017).

32. O. Diekmann, H. Heesterbeek, T. Britton, *Mathematical Tools for Understanding Infectious Disease Dynamics* (Princeton Univ. Press, 2012), vol. 7.

33. F. Miura, K. Y. Leung, D. Klinkenberg, K. E. C. Ainslie, J. Wallinga, Optimal vaccine allocation for COVID-19 in the Netherlands: A data-driven prioritization. *PLOS Comput. Biol.* **17**, e1009697 (2021).

34. J. Wallinga, M. van Boven, M. Lipsitch, Optimizing infectious disease interventions during an emerging epidemic. *Proc. Natl. Acad. Sci. U.S.A.* **107**, 923–928 (2010).

35. R. Durrett, *Random Graph Dynamics* (Cambridge Univ. Press, 2006), http://dx.doi.org/10.1017/cbo9780511546594.

36. K. Prem, K. van Zandvoort, P. Klepac, R. M. Eggo, N. G. Davies, Centre for the Mathematical Modelling of Infectious Diseases COVID-19 Working Group, A. R. Cook, M. Jit, Projecting contact matrices in 177 geographical regions: An update and comparison with empirical data for the COVID-19 era. *PLOS Comput. Biol.* **17**, e1009098 (2021).

37. O. Diekmann, J. A. P. Heesterbeek, M. G. Roberts, The construction of next-generation matrices for compartmental epidemic models. *J. R. Soc. Interface* **7**, 873–885 (2010).

38. M. Stein, Large sample properties of simulations using Latin hypercube sampling. *Technometrics* **29**, 143–151 (1987).

39. S. Blumberg, J. O. Lloyd-Smith, Inference of R(0) and transmission heterogeneity from the size distribution of stuttering chains. *PLOS Comput. Biol.* **9**, e1002993 (2013).

40. J. O. Lloyd-Smith, S. J. Schreiber, P. E. Kopp, W. M. Getz, Superspreading and the effect of individual variation on disease emergence. *Nature* **438**, 355–359 (2005).

41. E. M. Beer, V. B. Rao, A systematic review of the epidemiology of human monkeypox outbreaks and implications for outbreak strategy. *PLOS Negl. Trop. Dis.* **13**, e0007791 (2019).

42. H. Murayama, A. Nishi, A. Endo, Different time scales used for sexual partner surveys pose a challenge in modelling dynamics of sexually transmitted infections. medRxiv 23300526 [Preprint] (2023). https://doi.org/10.1101/2023.12.25.23300526.

43. H. Adler, S. Gould, P. Hine, L. B. Snell, W. Wong, C. F. Houlihan, J. C. Osborne, T. Rampling, M. B. Beadsworth, C. J. Duncan, J. Dunning, T. E. Fletcher, E. R. Hunter, M. Jacobs, S. H. Khoo, W. Newsholme, D. Porter, R. J. Porter, L. Ratcliffe, M. L. Schmid, M. G. Semple, A. J. Tunbridge, T. Wingfield, N. M. Price, M. Abouyannis, A. al-Balushi, S. Aston, R. Ball, N. J. Beeching, T. J. Blanchard, F. Carlin, G. Davies, A. Gillespie, S. R. Hicks, M. C. Hoyle, C. Ilozue, L. Mair, S. Marshall, A. Neary, E. Nsutebu, S. Parker, H. Ryan, L. Turtle, C. Smith, J. van Aartsen, N. F. Walker, S. Woolley, A. Chawla, I. Hart, A. Smielewska, E. Joekes, C. Benson, C. Brindley, U. das, C. K. Eyton-Chong, C. Gnanalingham, C. Halfhide, B. Larru, S. Mayell, J. McBride, C. Oliver, P. Paul, A. Riordan, L. Sridhar, M. Storey, A.

Abdul, J. Abrahamsen, B. Athan, S. Bhagani, C. S. Brown, O. Carpenter, I. Cropley, K. Frost, S. Hopkins, J. Joyce, L. Lamb, A. Lyons, T. Mahungu, S. Mepham, E. Mukwaira, A. Rodger, C. Taylor, S. Warren, A. Williams, D. Levitt, D. Allen, J. Dixon, A. Evans, P. McNicholas, B. Payne, D. A. Price, U. Schwab, A. Sykes, Y. Taha, M. Ward, M. Emonts, S. Owens, A. Botgros, S. T. Douthwaite, A. Goodman, A. Luintel, E. MacMahon, G. Nebbia, G. O'Hara, J. Parsons, A. Sen, D. Stevenson, T. Sullivan, U. Taj, C. van Nipsen tot Pannerden, H. Winslow, E. Zatyka, E. Alozie-Otuka, C. Beviz, Y. Ceesay, L. Gargee, M. Kabia, H. Mitchell, S. Perkins, M. Sasson, K. Sehmbey, F. Tabios, N. Wigglesworth, E. J. Aarons, T. Brooks, M. Dryden, J. Furneaux, B. Gibney, J. Small, E. Truelove, C. E. Warrell, R. Firth, G. Hobson, C. Johnson, A. Dewynter, S. Nixon, O. Spence, J. J. Bugert, D. E. Hruby, Clinical features and management of human monkeypox: A retrospective observational study in the UK. *Lancet Infect. Dis.* **22**, 1153–1162 (2022).

44. Y. D. Jeong, W. Hart, R. N. Thompson, M. Ishikane, T. Nishiyama, H. Park, N. Iwamoto, A. Sakurai, M. Suzuki, K. Aihara, K. Watashi, E. O. D. Op de Coul, N. Ohmagari, J. Wallinga, S. Iwami, F. Miura, Modelling the effectiveness of an isolation strategy for managing mpox outbreaks with variable infectiousness profiles. *Nat. Commun.* **15**, 7112 (2024).

45. RDC-Institut National de la Statistique, École de Santé Publique de Kinshasa et ICF, "RDC, Enquête Démographique et de Santé 2023–24: Rapport des indicateurs clés" (RDC-Institut National de la Statistique, École de Santé Publique de Kinshasa et ICF, Kinshasa, RDC et Rockville, MD, USA, 2024).

46. UNAIDS, HIV prevention among adolescent girls and young women. [Preprint] (2016). https://unaids.org/sites/default/files/media_asset/UNAIDS_HIV_prevention_among_adolescent_girls_and_young_women.pdf.

47. M. Prochazka, P. Vinti, A. Hoxha, A. Seale, A. Mozalevskis, R. Lewis, R. M. Sagastume, M. Scherzer, L. Dore, M. Doherty, Temporary adaptations to sexual behaviour during the mpox outbreak in 23 countries in Europe and the Americas: Findings from a retrospective cross-sectional online survey. *Lancet Infect. Dis.* **24**, 1309–1318 (2024).

48. T. A. Ferede, A. G. Muluneh, A. Wagnew, A. D. Walle, Prevalence and associated factors of early sexual initiation among youth female in sub-Saharan Africa: A multilevel analysis of recent demographic and health surveys. *BMC Womens Health* **23**, 147 (2023).

49. A. W. Rimoin, P. M. Mulembakani, S. C. Johnston, J. O. Lloyd Smith, N. K. Kisalu, T. L. Kinkela, S. Blumberg, H. A. Thomassen, B. L. Pike, J. N. Fair, N. D. Wolfe, R. L. Shongo, B. S. Graham, P. Formenty, E. Okitolonda, L. E. Hensley, H. Meyer, L. L. Wright, J.-J. Muyembe, Major increase in human monkeypox incidence 30 years after smallpox vaccination campaigns cease in the Democratic Republic of Congo. *Proc. Natl. Acad. Sci. U.S.A.* **107**, 16262–16267 (2010).

50. M. Wayengera, Conflict drives mpox epidemic in eastern DRC. *Science* **386**, 1356 (2024).

51. T. Wawina-Bokalanga, S. Merritt, E. Kinganda-Lusamaki, D. Jansen, M. Halbrook, Á. O'Toole, E. Pukuta-Simbu, E. Hasivirwe Vakaniaki, R. Ola-Mpumbe, P. Kwete-Mbokama, P. Akil-Bandali, C. Kacita, A. Ponga-Museme, N. Mapenzi-Kashali, A. Amuri-Aziza, O. Tshiani-Mbaya, P. Paku-Tshambu, P. H. L. F. Dantas, T. De Block, E. Lokilo-Lofiko, C. Muswamba-Kayembe, J.-C. Makangara-Cigolo, G. Luakanda-Ndelemo, D. J. Kelvin, C. Pratt, A. Ayouba, S. Tessema, A. Mauro Rezende, L. E. Hensley, E. Delaporte, D. Mwamba, L. Subissi, L. Liesenborghs, N. A. Hoff, M. Peeters, N. Low, S. Ahuka-Mundeke, J.-J. Muyembe-Tamfum, A. W. Rimoin, J. Kindrachuk, K. Vercauteren, A. Rambaut, P. Mbala-Kingebeni, Epidemiology and phylogenomic characterisation of two distinct mpox outbreaks in Kinshasa, DR Congo, involving a new subclade Ia lineage: A retrospective, observational study. *Lancet* **406**, 63–75 (2025).

52. L. Angeli, C. P. Caetano, N. Franco, P. Coletti, C. Faes, G. Molenberghs, P. Beutels, S. Abrams, L. Willem, N. Hens, Assessing the role of children in the COVID-19 pandemic in Belgium using perturbation analysis. *Nat. Commun.* **16**, 2230 (2025).

53. H. Caswell, *Sensitivity Analysis: Matrix Methods in Demography and Ecology* (Springer, 2019).

54. J. Mossong, N. Hens, M. Jit, P. Beutels, K. Auranen, R. Mikolajczyk, M. Massari, S. Salmaso, G. S. Tomba, J. Wallinga, J. Heijne, M. Sadkowska-Todys, M. Rosinska, W. J.

Edmunds, Social contacts and mixing patterns relevant to the spread of infectious diseases. *PLOS Med.* **5**, e74 (2008).

55. T. Hoang, P. Coletti, A. Melegaro, J. Wallinga, C. G. Grijalva, J. W. Edmunds, P. Beutels, N. Hens, A systematic review of social contact surveys to inform transmission models of close-contact infections. *Epidemiology* **30**, 723–736 (2019).

56. V. Latora, A. Nyamba, J. Simpore, B. Sylvette, S. Diane, B. Sylvére, S. Musumeci, Network of sexual contacts and sexually transmitted HIV infection in Burkina Faso. *J. Med. Virol.* **78**, 724–729 (2006).

57. J. N. Inungu, B. F. N. Kandala, A. M. Atungale, Prevalence of HIV and syphilis and related risk behaviors among female sex workers in the Democratic Republic of the Congo. *Eur. J. Environ. Public Health* **6**, em0114 (2022).

58. F. Miura, J. A. Backer, G. van Rijckevorsel, R. Bavalia, S. Raven, M. Petrignani, K. E. C. Ainslie, J. Wallinga, for the Dutch Mpox Response Team, B. van Benthem, D. Brandwagt, H. Bos, C. van Bokhoven-Rombouts, L. Bovée, C. P. Rovers, B. van Cleef, A. P. van Dam, R. van Dael, A. A. van der Eijk, P. Ellerbroek, C. van Ewijk, E. Franz, C. GeurtsvanKessel, J. van der Giessen, H. Götz, J. M. W. Häger, S. van den Hof, E. Hoornenborg, P. Hintaran, J. de Jonge, R. Joosten, M. Koopmans, K. Kosterman, J. Lange, T. Leenstra, D. Ooms, D. Oorsprong, E. op de Coul, D. Reurings, G. van Rijckevorsel, G. J. Sips, S. F. de Stoppelaar, A. Vollaard, B. Voordouw, H. Vennema, H. J. C. de Vries, K. Ellen Veldkamp, K. Weijdema, G. Westerhuis, M. J. M. te Wierik, M. R. A. Welkers, T. Waegemaekers, J. Wallinga, P. Zantkuijl, Time scales of human mpox transmission in the Netherlands. *J. Infect. Dis.* **229**, 800–804 (2024).

59. I. Brosius, C. Van Dijck, J. Coppens, L. Vandenhove, E. Bangwen, F. Vanroye, J. Verschueren, ITM MPOX Study Group, S. Zange, J. Bugert, J. Michiels, E. Bottieau, P. Soentjens, J. van Griensven, C. Kenyon, K. K. Ariën, M. Van Esbroeck, K. Vercauteren, L. Liesenborghs, Presymptomatic viral shedding in high-risk mpox contacts: A prospective cohort study. *J. Med. Virol.* **95**, e28769 (2023).

60. H. Kim, R. Kwon, H. Lee, S. W. Lee, M. Rahmati, A. Koyanagi, L. Smith, M. S. Kim, G. F. López Sánchez, D. Elena, S. G. Yeo, J. I. Shin, W. Cho, D. K. Yon, Viral load dynamics and shedding kinetics of mpox infection: A systematic review and meta-analysis. *J. Travel Med.* **30**, taad111 (2023).

61. C. Diaz Brochero, L. C. Nocua-Báez, J. A. Cortes, K. Charniga, A. Buitrago-Lopez, Z. M. Cucunubá, Decoding mpox: A systematic review and meta-analysis of the transmission and severity parameters of the 2022-2023 global outbreak. *BMJ Glob. Health* **10**, e016906 (2025).

62. L. Ponce, N. M. Linton, W. H. Toh, H.-Y. Cheng, R. N. Thompson, A. R. Akhmetzhanov, J. Dushoff, Incubation period and serial interval of mpox in 2022 global outbreak compared with historical estimates. *Emerg. Infect. Dis.* **30**, 1173–1181 (2024).

63. United Nations, Department of Economic and Social Affairs, Population Division, World Population Prospects 2024, Online Edition (2024), https://population.un.org/wpp/.

64. V. Marziano, G. Guzzetta, I. Longini, S. Merler, Epidemiologic quantities for monkeypox virus clade I from historical data with implications for current outbreaks, Democratic Republic of the Congo. *Emerg. Infect. Dis.* **30**, 2042–2046 (2024).

65. Z. Jezek, B. Grab, M. V. Szczeniowski, K. M. Paluku, M. Mutombo, Human monkeypox: Secondary attack rates. *Bull. World Health Organ.* **66**, 465–470 (1988).

66. S. Colombe, S. Funke, A. Koch, M. Haverkate, S. Monge, A.-S. Barret, A. Vaughan, S. Hahné, C. van Ewijk, H.-D. Emborg, S. von Schreeb, A. Díaz, C. Olmedo, L. Zanetti, D. Levy-Bruhl, L. A. de Sousa, J. Hagan, N. Nicolay, R. Pebody, Effectiveness of historical smallpox vaccination against mpox clade II in men in Denmark, France, the Netherlands and Spain, 2022. *Euro Surveill.* **29**, 2400139 (2024).

67. B. K. Titanji, A. Eick-Cost, E. S. Partan, L. Epstein, N. Wells, S. L. Stahlman, P. Devineni, B. Munyoki, S. Pyarajan, A. Balajee, J. Smith, C. W. Woods, M. Holodniy, V. J. Davey, R. A. Bonomo, Y. Young-Xu, V. C. Marconi, Effectiveness of smallpox vaccination to prevent mpox in military personnel. *N. Engl. J. Med.* **389**, 1147–1148 (2023).

68. E. Vynnycky, R. White, *An Introduction to Infectious Disease Modelling* (Oxford Univ. Press, 2010).
